# Acute and chronic effects of treatment with mesenchymal stromal cells on LPS-induced pulmonary inflammation, emphysema and atherosclerosis development

**DOI:** 10.1371/journal.pone.0183741

**Published:** 2017-09-14

**Authors:** P. Padmini S. J. Khedoe, Stan de Kleijn, Annemarie M. van Oeveren-Rietdijk, Jaap J. Plomp, Hetty C. de Boer, Melissa van Pel, Patrick C. N. Rensen, Jimmy F. P. Berbée, Pieter S. Hiemstra

**Affiliations:** 1 Dept. of Pulmonology, Leiden University Medical Center, Leiden, The Netherlands; 2 Dept. of Medicine, Div. of Endocrinology, Leiden University Medical Center, Leiden, The Netherlands; 3 Dept. of Medicine, Div. of Nephrology, Leiden University Medical Center, Leiden, The Netherlands; 4 Einthoven Laboratory for Experimental Vascular Medicine, Leiden University Medical Center, Leiden, The Netherlands; 5 Dept. of Neurology, Leiden University Medical Center, Leiden, The Netherlands; 6 Dept. of Immunohematology and Blood Transfusion, Leiden University Medical Center, Leiden, The Netherlands; Institute of Lung Biology and Disease (iLBD), Helmholtz Zentrum München, GERMANY

## Abstract

**Background:**

COPD is a pulmonary disorder often accompanied by cardiovascular disease (CVD), and current treatment of this comorbidity is suboptimal. Systemic inflammation in COPD triggered by smoke and microbial exposure is suggested to link COPD and CVD. Mesenchymal stromal cells (MSC) possess anti-inflammatory capacities and MSC treatment is considered an attractive treatment option for various chronic inflammatory diseases. Therefore, we investigated the immunomodulatory properties of MSC in an acute and chronic model of lipopolysaccharide (LPS)-induced inflammation, emphysema and atherosclerosis development in APOE*3-Leiden (*E3L*) mice.

**Methods:**

Hyperlipidemic *E3L* mice were intranasally instilled with 10 μg LPS or vehicle twice in an acute 4-day study, or twice weekly during 20 weeks Western-type diet feeding in a chronic study. Mice received 0.5x10^6^ MSC or vehicle intravenously twice after the first LPS instillation (acute study) or in week 14, 16, 18 and 20 (chronic study). Inflammatory parameters were measured in bronchoalveolar lavage (BAL) and lung tissue. Emphysema, pulmonary inflammation and atherosclerosis were assessed in the chronic study.

**Results:**

In the acute study, intranasal LPS administration induced a marked systemic IL-6 response on day 3, which was inhibited after MSC treatment. Furthermore, MSC treatment reduced LPS-induced total cell count in BAL due to reduced neutrophil numbers. In the chronic study, LPS increased emphysema but did not aggravate atherosclerosis. Emphysema and atherosclerosis development were unaffected after MSC treatment.

**Conclusion:**

These data show that MSC inhibit LPS-induced pulmonary and systemic inflammation in the acute study, whereas MSC treatment had no effect on inflammation, emphysema and atherosclerosis development in the chronic study.

## Introduction

Chronic Obstructive Pulmonary Disease (COPD) is defined as a pulmonary disorder with chronic lung inflammation and is often accompanied by comorbidities including cardiovascular disease (CVD) [1]. COPD patients have an increased risk to develop CVD compared to matched controls, even after correction for common risk factors [1, 2]. COPD is mainly caused by cigarette smoking and is characterized by irreversible progressive airflow limitation and an abnormal pulmonary inflammatory response to noxious particles or gases [1]. COPD is also often accompanied by elevated levels of circulating acute phase proteins and pro-inflammatory cytokines, including serum amyloid A (SAA), C-reactive protein (CRP), interleukin-6 (IL-6) and tumor necrosis factor-α (TNF-α) [3]. Systemic inflammation, next to hyperlipidemia, also contributes to atherosclerosis development, which is the most important underlying cause of CVD [4]. Also other factors including cigarette smoking, oxidative stress and lipopolysaccharide (LPS—a component of cigarette smoke and gram-negative bacteria), contribute both to COPD [1] as well as atherosclerosis development [5]. Low-grade systemic inflammation in COPD, triggered by cigarette smoke and repeated respiratory microbial exposure, is suggested to link COPD and CVD [3, 6, 7]. Especially during exacerbations, when a sudden worsening of COPD symptoms occurs which is accompanied by and likely partly triggered by respiratory infections, there is an increased risk for hospitalization and CVD morbidity and mortality [1].

Current treatment of COPD patients consists of inhaled bronchodilator and anti-inflammatory therapy [3], to which lipid-lowering treatment (*e*.*g*. statins), antihypertensive agents or β-blockers are added to prevent or treat CVD [6, 8]. Although some studies show that statin therapy may be beneficial also for COPD symptoms [3, 9], it is not known whether this treatment approach is most optimal for COPD patients with CVD. The symptoms of both diseases are treated as separate modalities and do not take the possible interactions between COPD and CVD into account. Therefore, there is a need for new combined treatment options, which target both COPD and atherosclerosis. A promising therapeutic strategy is treatment with mesenchymal stromal cells (MSC). MSC inhibit pro-inflammatory responses through induction of immunomodulatory cells (regulatory cells) and inhibition of pro-inflammatory cells. Furthermore, MSC may contribute to regeneration of damaged tissue [10, 11]. Although it has been suggested that MSC have long-term regenerative and immunomodulatory effects, their precise mechanism of action is unknown. After intravenous injection, viable MSC can be traced back in the lung 24 h after infusion, whereas other organs are devoid of viable MSC [12, 13], suggesting that MSC mostly exert their immunomodulatory and protective effect through paracrine mechanisms [14].

Administration of MSC has shown beneficial effects in several inflammatory diseases, including Crohn’s disease, Graft-versus-host disease and rejection after organ transplantation [10, 11, 15]. Furthermore, several preclinical studies using animal models of COPD have shown that treatment with MSC reduces pulmonary inflammation and emphysema development [16–20]. So far, MSC treatment has been evaluated in a few clinical trials in COPD and was found to be safe, although currently little effects on disease outcomes were observed [21–23]. Also several mouse models of atherosclerosis show that treatment with MSC reduces CVD [24–26]. Since current treatment of COPD patients with CVD is suboptimal, the aim of this study was to investigate the immunomodulatory properties of MSC on pulmonary and systemic inflammation, emphysema and atherosclerosis in an acute and chronic murine model of LPS-induced pulmonary inflammation using hyperlipidemic APOE*3-Leiden (*E3L*) mice, which develop diet-induced atherosclerosis [27, 28].

## Materials and methods

### Animals and experimental procedure

All animal experiments described in this paper were approved by the Institutional Ethical Committee on Animal Care and Experimentation of the Leiden University Medical Center (LUMC, Leiden, The Netherlands).

Mice were housed under standard conditions with a 12-hour light/dark cycle and had *ad libitum* access to food and water. Female *E3L* mice (LUMC, Leiden, The Netherlands) of 10–12 weeks of age were fed a Western-type diet (WTD) containing 15% (w/w) cacao butter, 1% (w/w) corn oil (diet-T; Hope Farms, Woerden, The Netherlands) and 0.4% (w/w) cholesterol. Mice were fed the WTD during 3 weeks of run-in after which they were matched based on age, body weight and plasma lipid levels (S1 Fig—acute study; S2 Fig—chronic study) and divided over four groups: vehicle, MSC, LPS and LPS+MSC (Table 1).

**Table 1:** Groups and groups sizes.

	Acute study (n)	Chronic study (n)
Vehicle	8	14
MSC	8	13
LPS	10	14
LPS+MSC	10	14

#### Acute study

We performed two studies: an *acute* study (4 days–for study outline see S1 Fig) and a *chronic* study (20 weeks–for study outline see S2 Fig). In the *acute* study we determined the effect and feasibility of MSC treatment on LPS-induced acute pulmonary and systemic inflammation. To this purpose, 10 μg LPS (serotype 055:B5 *Escherichia coli* LPS, Sigma-Aldrich, Zwijndrecht, The Netherlands) in 50 μl sterile PBS was administered intranasally twice (on day 1 and 3). Control mice received 50 μl sterile PBS (vehicle). MSC (0.5x10^6^ cells in 200 µl PBS) or 200 μl sterile PBS as control was administered intravenously by tail vein injection on day 1 and 2. Blood was collected after LPS administration to determine the systemic IL-6 response. Mice were sacrificed on day 4 as described below.

#### Chronic study

In the *chronic* study (for study outline see S2 Fig) we determined the effect of MSC treatment on LPS-induced chronic pulmonary and systemic inflammation, emphysema and atherosclerosis development. To this purpose, 10 μg LPS (serotype 055:B5 *Escherichia coli* LPS) in 50 μl sterile PBS was intranasally administered twice weekly, during 20 weeks WTD feeding to induce diet-induced atherosclerosis [27]. Control mice received 50 μl sterile PBS (vehicle). From week 14 onwards, mice received MSC intravenously by tail vein injection every other week (i.e. week 14, 16, 18 and 20). Blood was collected every 4 weeks to determine plasma lipid levels. Mice were sacrificed 24 h after the last LPS instillation.

#### Necropsy

In both studies mice were anesthetized by intraperitoneal injection of 6.25 mg/kg acepromazine (Alfasan, Woerden, The Netherlands), 6.25 mg/kg midazolam (Roche, Mijdrecht, The Netherlands), and 0.31 mg/kg fentanyl (Janssen-Cilag, Tilburg, The Netherlands). Subsequently, blood was collected through orbital puncture after which mice were sacrificed by cervical dislocation. Bronchoalveolar lavage (BAL) was performed by insertion of a tracheal cannula through which 2x 500 μl ice-cold PBS was infused and retrieved. Lung lavage was collected on ice for further processing. After BAL, the mice were perfused with ice-cold PBS through the heart to remove remaining blood from the circulation.

In the chronic study, half of the right lung was saved for flow cytometric analysis, and the other half was stored at -80°C. Left lungs were fixed *in situ* by gentle infusion of the fixative phosphate-buffered 4% formaldehyde (PFA) by a continuous-release pump under constant pressure (12 ml/h; 8 min) through a tracheal cannula. After excision, the lungs and heart were immersed in fresh fixative for a period of 24 h at 4°C. Other organs were isolated and stored at -80°C for further analysis.

### MSC isolation

In both studies, MSC were isolated from bone marrow of donor female *E3L* mice of 8–10 weeks of age and cultured as previously described [29]. In short, bone marrow from donor mice was flushed, the eluted cells were collected, washed and cultured in DMEM containing 100 μg/ml penicillin and 100 μg/ml streptomycin, 10% heat-inactivated FCS and L-glutamine (all from Gibco, Thermofisher Scientific). After 3 hours, non-adherent cells were removed and thereafter medium was refreshed every 8 hours, up to 72 hours of initial culture, after which medium was refreshed 2 times a week, till confluence was reached. The MSCs that were used throughout this study were of passage 4–7; for *in vivo* MSC treatment cells of passage 4–6 were used. Cells were frozen in culture medium containing 10% DMSO (VWR, The Netherlands) till use for *in vitro* characterization or *in vivo* administration. MSC were thawed and cultured overnight, before *in vivo* administration. For *in vitro* characterization, MSC were cultured until confluent, after which they were washed, trypsinized and phenotyped using the antibodies listed in Table 2. Immune regulatory functions of MSC were determined *in* *vitro* by stimulating mouse splenocytes with anti-CD3/CD28 microbeads (Life Technologies, Bleiswijk, The Netherlands) and culturing them in the presence or absence of MSC for 5 days at 37°C and 5% CO_2_. Next, the cultures were incubated for 16 hours with ^3^H-thymidine, and incorporation was measured as a percentage of the control to determine the effect of MSC on T cell proliferation. To determine the adipogenic and osteogenic differentiation capacity of the isolated MSC, MSC were cultured for 21 days in culture medium containing either 10^−6^ M dexamethasone, 5 μM insulin, 0.5 mM 3-isobutyl-1-methylxanthine and 100 μM indomethacin (adipogenic) or 10^−8^ M dexamethasone, 10 mM β-glycerol-phosphate, and 5 μg/mL ascorbic acid (osteogenic) (all from Sigma-Aldrich Chemie BV, Zwijndrecht, The Netherlands). Oil-red-O (Sigma-Aldrich) was used to determine adipogenic differentiation, whereas alkaline phosphatase (Vector laboratories) and Alizarin Red (Sigma-Aldrich) were used to determine osteogenic differentiation and mineralization, resp (21).

**Table 2:** Antibodies used for MSC characterization.

Marker	Cell type	Source
TER119	Erythrocytes	TER119, BD Biosciences
CD31	Endothelial cells	MEC 13.3, BD Biosciences
CD45.2	Leukocyte Common Antigen	104, BD Biosciences
CD29		HMb1-1, BD Biosciences
Sca-1	multipotent hematopoietic stem cells	D7, BD Biosciences
CD105		MJ7/28, BD Biosciences
CD44		IM7, BD Biosciences
CD106		429, BD Biosciences

### Pulmonary function measurements

Total respiratory amplitude and respiratory rate were assessed at 12 and 20 weeks in the chronic study with non-invasive whole-body plethysmography (RM-80, Columbus Instruments, Columbus, OH, USA). The total respiratory amplitude was calculated from the measured peak-to-peak signals and reflects the tidal volume. Flow-derived parameters of breath amplitude and frequency were collected for and averaged over 2 min per mouse. The signal was digitized using a Digidata 1440A interface (Axon Instruments/Molecular Devices, Union City, CA, USA) and analyzed with the event detection feature of Clampfit 10.4 (Axon Instruments/Molecular Devices). Peak amplitude values were corrected for body weight.

### Plasma lipids and systemic inflammation analysis

Plasma total cholesterol (TC), triglyceride (TG) and phospholipid (PL) levels were determined using enzymatic kits from Roche Molecular Biochemicals (Woerden, The Netherlands) according to the manufacturer’s protocols. Plasma IL-6 levels were determined in the acute study using a murine IL-6 kit (BD Biosciences Pharmingen) according to the manufacturer’s instructions. In the chronic study, serum amyloid A (SAA) and IL-6 levels (in serum) and KC levels (in BAL) were determined using murine kits for SAA, IL-6 and CXCL1/KC kit, resp. (R&D systems) according to manufacturer’s instructions.

### FACS analysis of whole blood in the chronic study

In the chronic study, blood was collected at T = 12 and T = 19 weeks in EDTA-coated tubes and whole blood was analyzed by Sysmex KX-21N™ Automated Hematology Analyzer and FACS. White blood cell counts (WBC, n ×10^6^/ml), red blood cell counts (RBC, n ×10^9^/ml), platelets (PLT, n ×10^6^/ml), haematocrit (HCT, %/%), and haemoglobin (HGB, mmol/l) were obtained using Sysmex. For FACS analysis, whole blood was incubated 30 min with antibodies as listed in Table 3. Doublets were excluded and as a control, whole blood was incubated with the included cocktail of isotype controls, to identify the threshold for lineage-positivity. Data was analyzed with BD FACSDiva software (version 6.0, BD Biosciences).

**Table 3:** Antibodies used for FACS analysis of whole blood.

Marker	Label	Cell type	Source
Ly6G	eFluor450	neutrophils	#48–5931, eBioscience
CD115	Biotin	monocyte/macrophage	#13–1152, eBioscience
CD11b	APC	Myeloid + NK-cells	#553312, BD Pharmingen
Ly6C (MP20)	Alexa488	monocyte/macrophage	#MCA2389A488, Serotec/Bioconnect
B220 (CD45R)	APC-eFluor780	B and T-cells	#47–0452, eBioscience
CCR2	PE	monocyte/macrophage	#FAB5538P, R&D Systems
Lineage	APC	hematopoietic lineage cells	#558074, BD Pharmingen
Sca (Ly6A/E)	FITC	multipotent hematopoietic stem cells	#553335, BD Pharmingen
c-kit (CD117)	Biotin	hematopoietic progenitor cells	#13–1171, eBioscience
Flk (CD309)	PE	hematopoietic progenitor cells	#555308, BD Pharmingen

### BAL cytospin preparation

Collected BAL fluid was centrifuged at 5,000 rpm for 5 min at room temperature (RT) in both studies. Supernatant was collected and stored at -80°C for further analysis. Pellets were resuspended in PBS with 1% bovine serum albumin (BSA) (A7030, Sigma-Aldrich, The Netherlands). Cells were counted using the Countess cell counter (Thermofisher Scientific, Bleiswijk, The Netherlands) and cytospins were prepared at a concentration of 0.3x10^6^ cells/ml on SuperFrost glass slides (Thermo Scientific, The Netherlands). Cytospins were air-dried and a Diff-quick staining (Kit RAL555, Reactifs-RAL, Cellpath, England) was performed, after which 300–600 cells were counted using light-microscopy. Macrophages, neutrophils, lymphocytes and epithelial cells were counted based on morphology. Cells were expressed as number of cells per/ml.

### BAL and lung tissue processing for flow cytometry

In the chronic study, lung tissue and BAL cells were prepared for flow cytometry analysis. Lung tissue was incubated with collagenase (1 mg/ml, 234153, Clostridium histolyticum, Calbiochem, Germany) and DNase (2 U/ml, D4263, Sigma-Aldrich, USA) in PBS, after which a single cell suspension was prepared. Cells were then centrifuged for 5 min at 1,500 rpm at 4°C. Red blood cells were removed using shock buffer (containing 8.3 g/l NH_4_Cl, 1 g/l KHCO_3_ and 37 mg/l EDTA). Cells were washed and centrifuged for 5 min at 1,500 rpm at 4°C. The pellet was resuspended and plated in a 96 wells plate (Nunc MaxiSorp®, eBioscience) after which the cells were stained with the live/dead staining Aqua (Life technologies, Bleiswijk, The Netherlands). After fixing the cells in a 1.9% PFA solution for 15 min at RT, they were washed and resuspended in FACS buffer, after which they were stained for flow cytometric analysis. Antibodies that were used are listed in Table 4. Mouse FcγRII/III-binding inhibitor (kindly provided by Louis Boon, Bioceros) was added to the antibody mix. Measurements were performed on a FACSCanto II (BD Bioscience, San Jose, CA) and analyzed using FlowJo (v7.6.5) software (Tree Star, Ashland, OR).

**Table 4:** Antibodies used for FACS analysis of BAL cells and lung tissue.

Marker	Label	Cell type	Source
Ly-6G and Ly-6C (GR1)	FITC	Neutrophils	RB6-8C5, BD Biosciences
SiglecF	PE	Eosinophils	E50-2440, BD Biosciences
CD3	PerCP-efluor 710	T-cell	17A2, eBioscience
CD11b	PE Cy7	Multiple	M1/70, eBioscience
MHCII (I-A/I-E	APC	Dendritic cells	M5/114.15.2, eBiosicience
CD45R (B220)-	APC-efluor 780	B-cell	RA3-6B2, eBioscience
CD11c	Horizon V450	Multiple	HL3, BD Biosciences
Aqua	viability		
Mouse FcγRII/III-binding inhibitor			Provided by Louis Boon, Bioceros

### Histological analyses of the lungs

Lungs were processed for paraffin embedding and cut into 5μm coronal sections. Tissue samples were stained with hematoxylin-eosin (HE, Klinipath). For immunohistochemical staining of lungs obtained from the acute study, slides were pretreated with 1% H_2_O_2_ in methanol to block endogenous peroxidase activity. Sections were incubated overnight with anti-myeloperoxidase (MPO) (1:1500, Thermo Fisher Scientific, Runcorn, United Kingdom) to detect neutrophils in the lung, and washed with PBS/0.05% Tween-20. Anti-rabbit EnVision-HRP was added and incubated for 30 min. Subsequently, slides were washed with PBS/0.05% Tween-20 and peroxidase-streptavidin was applied to the slides and incubated for 30 min. After washing with PBS/0.05% Tween-20, Nova Red substrate (Vector Laboratories Inc., Burlingame, CA) was added and incubated for 5 min. Slides were rinsed and counterstained with hematoxylin. MPO-positive cells were counted in 10 non-overlapping fields per mouse using a Olympus CX41 microscope (400x magnification) and averaged per group.

To assess air space enlargement in the chronic study, mean linear intercept (MLI) and air/tissue (AT) ratio were quantified in a blinded fashion by superimposing a NGW2 line grid (Olympus) with 21 lines and 42 points on the images of HE-stained lung sections at a magnification of 200x using an Olympus CX41 microscope as described previously [28]. To calculate the MLI, the number of intersections between the lines of the grid and the alveolar walls was quantified for each mouse in 10 non-overlapping fields. To determine the AT ratio, in the number of points in alveolar space was counted 10 non-overlapping fields and divided by the total number of 42 points. Additionally, Azan trichrome staining was performed according to the manufacturer’s protocol (Klinipath, Duiven, The Netherlands). The stained slides were evaluated by The Ashcroft method [30] to assess lung fibrosis, and lung tissue was categorized as follows: grade 0, normal lung; grade 1, minimal fibrous thickening of the alveolar or bronchiolar walls; grade 3, moderate thickening of the walls without obvious damage to lung architecture; grade 5, increased fibrosis with definite damage to the lung structure and the formation of fibrous bands or small fibrous masses; grade 7, severe distortion of the structure and large fibrous areas; grade 8, total fibrous obliteration of fields. Inflammation was also semi-quantified in a similar manner: grade 1, no inflammation; grade 1, mild inflammation; grade 2 high inflammation and grade 3, severe inflammation [31]. Both semi-quantitative analyses were performed in 10 non-overlapping fields for each mouse.

### Histological analyses of the heart

At the end of the chronic study, hearts were isolated and fixed in phosphate-buffered 4% formaldehyde and processed for paraffin embedding. For quantification and classification of atherosclerosis, the hearts were cross-sectioned (5 μm) throughout the entire aortic root area. Per mouse, 4 sections with 40 μm intervals were used for quantification of atherosclerotic lesion area and characterization of lesion severity by staining with hematoxylin-phloxine-saffron (HPS). Atherosclerotic lesions were categorized for severity, according to the guidelines of the American Heart Association, [32] adapted for mice. The segments were categorized into: 1) no lesions 2) mild (type I-III) and 3) severe (type IV-V) lesions [32].

For immunohistochemical staining, slides were pretreated with 1% H_2_O_2_ in methanol to block endogenous peroxidase activity. Sections were incubated overnight with rat anti-mouse MAC-3 antibody (1:50, BD Pharmingen, Breda, The Netherlands) to detect macrophages, after which they were washed with PBS/0.05% Tween-20. Biotinylated anti-rat EnVision-HRP (DAKO, The Netherlands) was added and incubated for 30 min. After washing with PBS/0.05% Tween-20, Nova Red substrate (Vector Laboratories Inc., Burlingame, CA) was added and incubated for 5 min. Slides were rinsed and counterstained with hematoxylin. Stained slides were then digitized using a Philips Ultra Fast Scanner (Philips Digital Pathology Solutions, Best, The Netherlands). Snap-shots of the aortic root area were taken using the Philips Image Management System and Philips IMS Application Server and Storage software (Philips Digital Pathology Solutions, Best, The Netherlands). Total lesion area and MAC-3 positive macrophage content in the lesions was quantified on the digital snap-shots using Image-J analysis software.

### Statistical analysis

Statistical significance of differences was assessed using one-way ANOVA or repeated measures analysis, followed by post-hoc analysis using Fisher’s LSD multiple comparison test. Differences at *p*<0.05 were regarded as statistically significant. Data are presented as means ± SEM.

## Results

### MSC characterization and *in vitro* immune regulatory functions

Isolated MSC were positive for the classical MSC markers, including Sca-1, CD29 and CD106, whereas hematopoietic lineage markers were absent (S3A Fig). Furthermore, immune regulatory capacity of the isolated MSC was investigated *in vitro* in a T cell proliferation assay. MSC dose-dependently inhibited T cell proliferation (S3B Fig). Furthermore, MSC were able to differentiate *in vitro* in adipocytes and osteoblasts (S3C Fig), indicating that the isolated MSC were functional. Together, this indicated that the cells used throughout this study were functional and phenotypically MSC.

### MSC treatment reduces pulmonary and systemic inflammation

**Fig 1:**
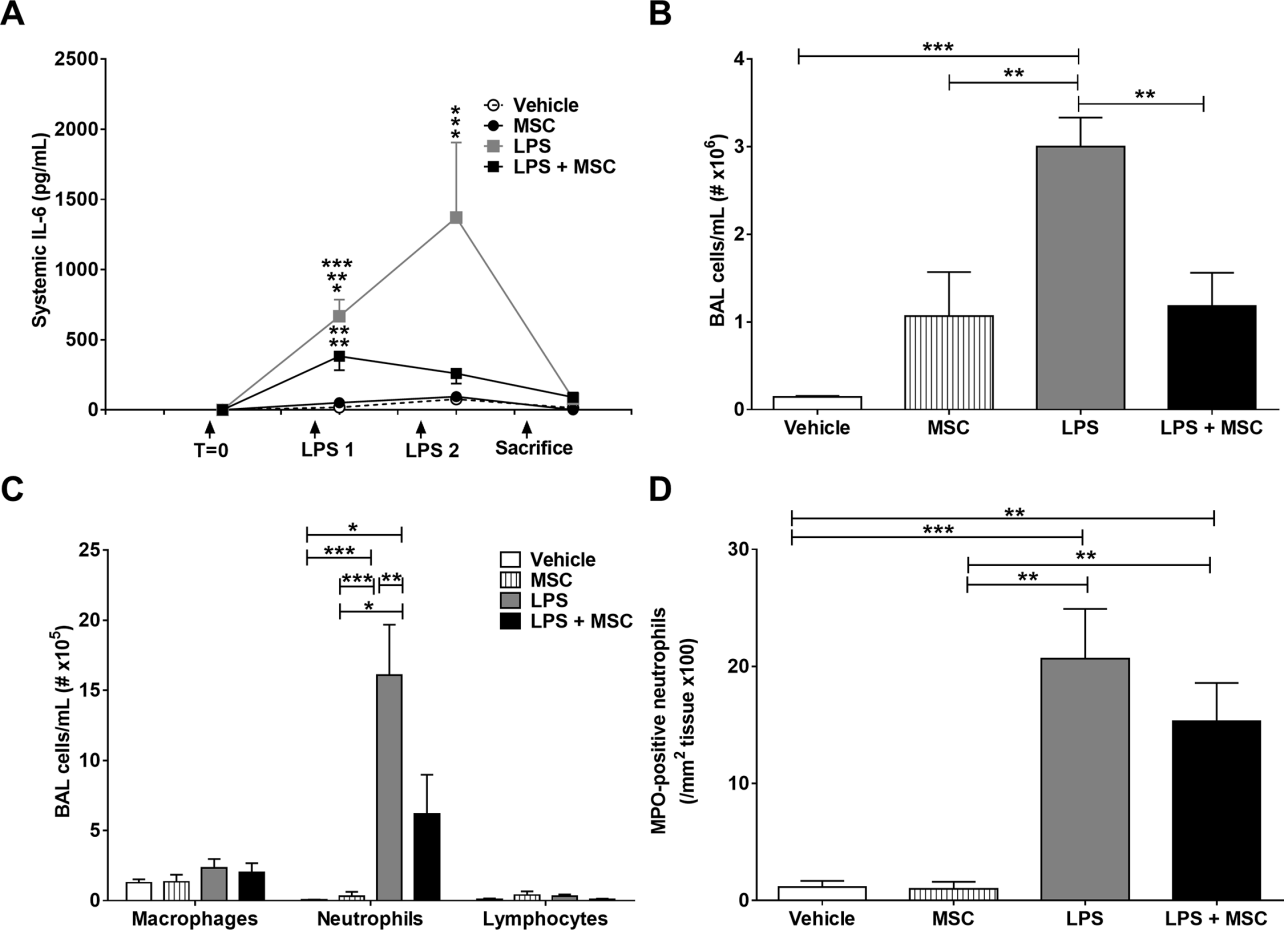
MSC treatment reduces inflammation in acute LPS-induced pulmonary inflammation. APOE*3-Leiden (*E3L*) mice fed a Western-type diet (WTD) were intranasally instilled with vehicle or 10 μg LPS on day 1 and 3. Mice received 0.5x10^6^ MSC 4 h and 24 h after the first LPS instillation. Blood was drawn 3 h after LPS instillation to measure systemic IL-6 response (A). Mice were sacrificed on day 4 after which BAL was collected and total (B) and differential (C) cell number was counted. The number of MPO-positive neutrophils in the lungs was determined immunohistochemically (D). Data are shown as mean±SEM; n = 12–15; *p<0.05, **p<0.01, ***p<0.001.

To examine the effect of intravenous MSC administration in the *acute* study, *E3L* mice were intranasally instilled with LPS on day 1 and 3. LPS induced a marked systemic IL-6 response on day 3, which was significantly reduced by MSC administration at 4 h and 24 h after the first LPS administration (Fig 1A). LPS increased total cell count (Fig 1B) and differential cell count (Fig 1C) in BAL, which was partly prevented by treatment with MSC, mostly due to reduced neutrophils numbers (Fig 1C). Neutrophils were increased in lung tissue sections after LPS instillation, whereas MSC treatment did not significantly alter this parameter (Fig 1D). Collectively, these data show that MSC treatment lowers systemic and pulmonary inflammation in a model of acute intranasal LPS-induced pulmonary inflammation.

### MSC administration does not affect chronic LPS-induced lowering in body weight, food intake and cholesterol levels

**Fig 2:**
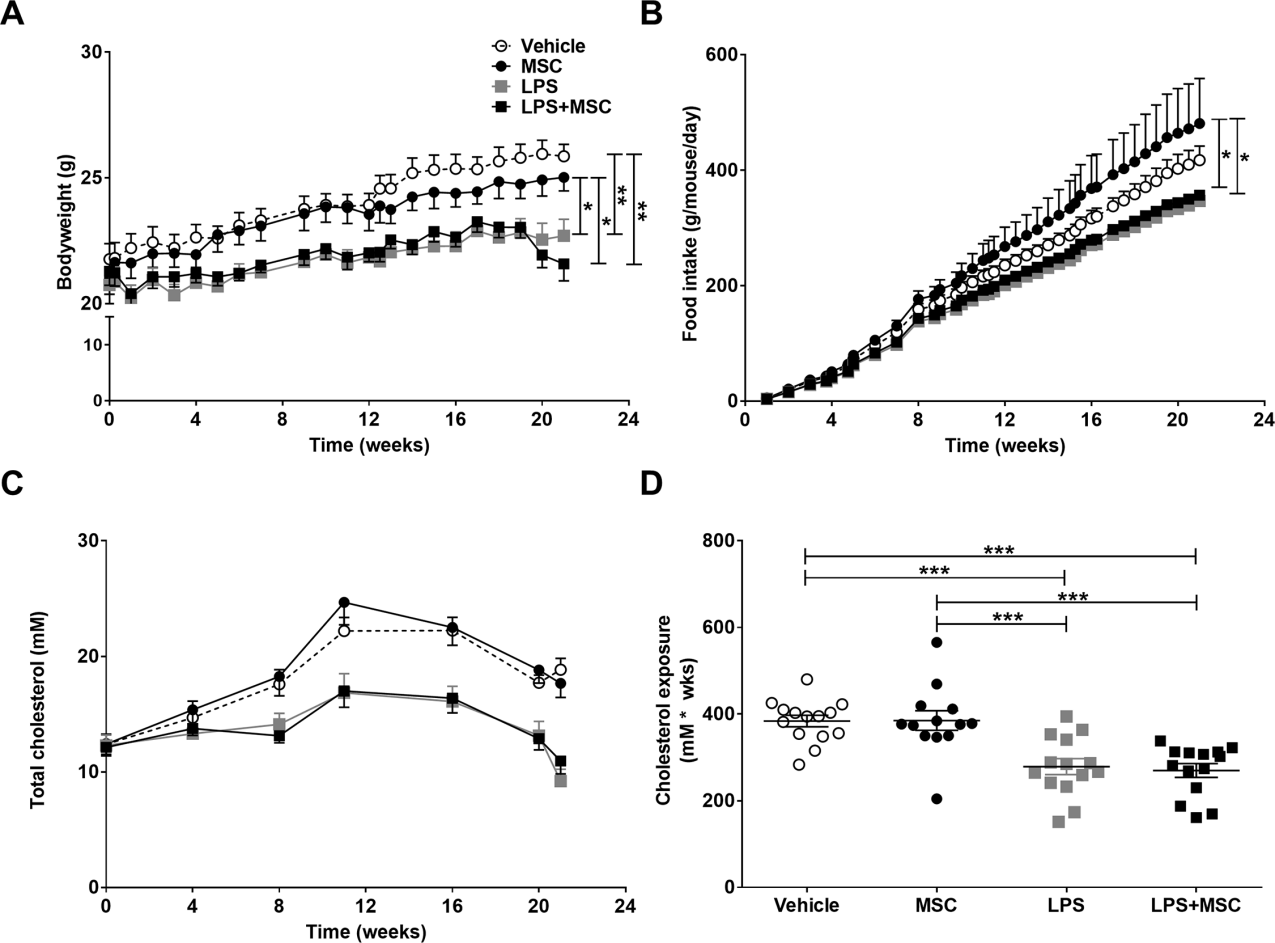
MSC treatment does not affect chronic LPS-induced lowering in body weight, food intake and cholesterol. E3L mice were intranasally instilled with vehicle or 10 μg LPS twice weekly during 20 weeks WTD feeding. Mice received vehicle or 0.5x10^6^ MSC in week 14, 16, 18 and 20. Bodyweight (A) and food intake (B) were monitored twice weekly during the study. Blood was drawn every 4 weeks to measure systemic cholesterol levels (C), which were used to calculate cholesterol exposure (D). Data are shown as mean±SEM; n = 12–15; *p<0.05, **p<0.01, ***p<0.001.

To examine the effect of MSC treatment in the *chronic* study, *E3L* mice were intranasally instilled with LPS twice weekly during 20 week WTD-feeding and MSC were intravenously administered in weeks 14, 16, 18 and 20. Mice exposed to intranasal LPS instillation, showed a lower body weight from the start of LPS instillation which persisted till the end of the study compared to vehicle instillation (Fig 2A). This effect was not restored by concomitant MSC treatment. The lowering in body weight in both LPS-treated groups was partly caused likely by a lower food intake, which was even significant compared to the mice that received MSC only (Fig 2B). Plasma cholesterol levels were lower upon LPS instillation (Fig 2C), possibly by reduced intake of the cholesterol-containing food, whereas MSC treatment did not restore the lowered cholesterol levels. This resulted in a lower cholesterol exposure in LPS-treated animals (Fig 2D).

**Fig 3:**
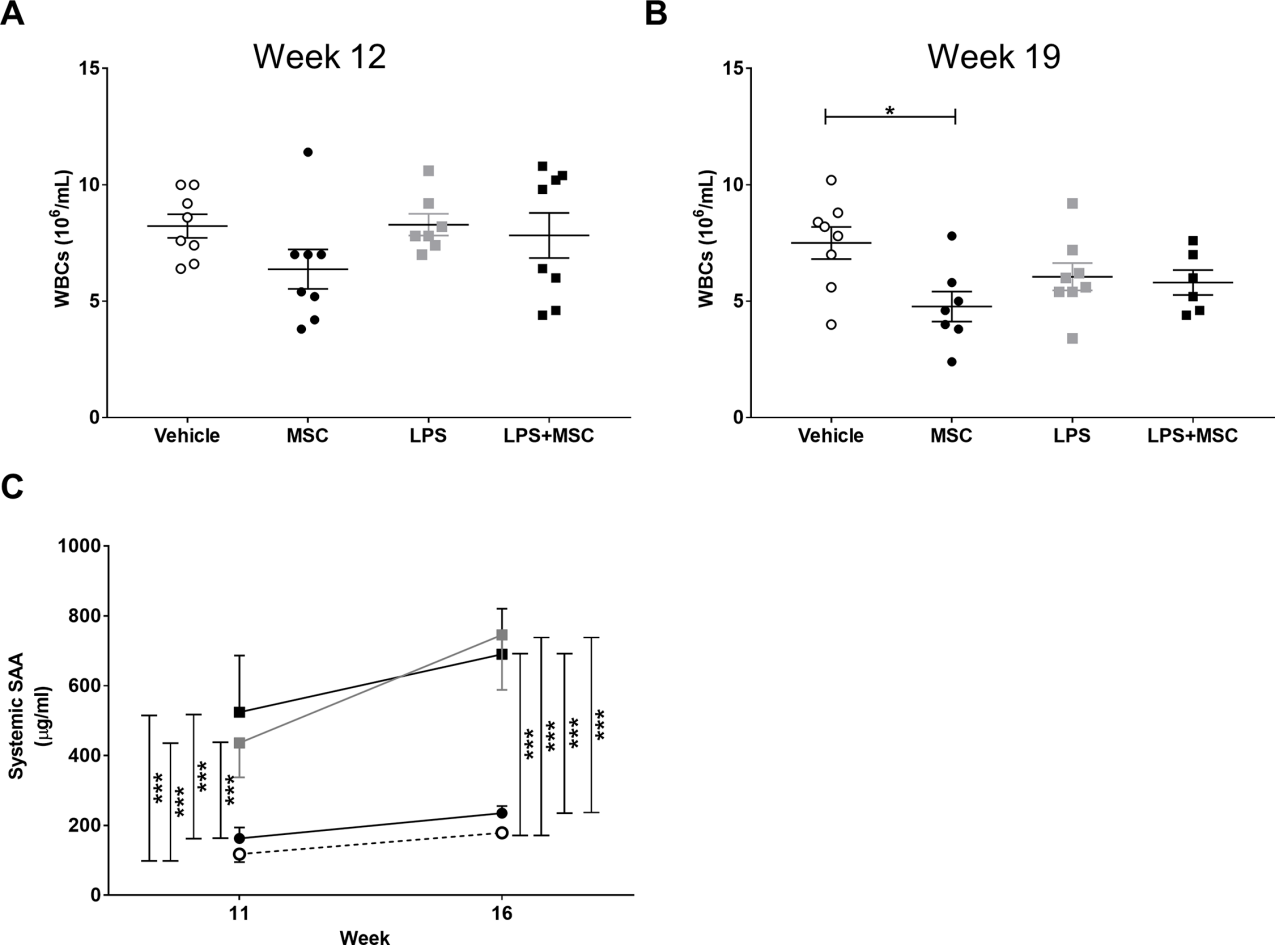
LPS exposure or MSC treatment do not affect blood leukocytes. E3L mice were intranasally instilled with vehicle or 10 μg LPS twice weekly during 20 weeks WTD feeding. Mice received vehicle or 0.5x10^6^ MSC in week 14, 16, 18 and 20. Blood was drawn at 12 and 19 weeks and blood leukocytes, including WBC counts (A and B) were measured using an automated cell counter (Sysmex) and FACS analysis. (C) Levels of SAA were measured in plasma of mice at week 11 and week 16 (before and after MSC treatment). Data for A and B are shown as mean±SEM; n = 6–8; *p<0.05. Data in C are shown as mean±SEM; n = 12–15; ***p<0.001.

At 12 weeks, just before start of MSC treatment, and at 19 weeks, blood was collected to measure the effect on circulating cells using an automated cell counter (Sysmex) and FACS analysis. WBC count was reduced at t = 19 weeks compared to t = 12 weeks in the mice that received MSC compared to mice that received vehicle only. Although there were differences between the groups already before start of the MSC treatment in different populations, there were no differences between the groups in any of the populations between the two time points, suggesting that there was no effect of MSC treatment on circulating leukocytes, although mice that received MSC only showed a decrease in leukocytes at week 19 (Fig 3A and 3B). Furthermore, systemic SAA (Fig 3C) and IL-6 (S4B Fig) levels were measured 24h after LPS instillation before and after the start of MSC treatment, at week 11 and 16 respectively. Mice that received intranasal LPS instillation showed elevated levels of SAA at both time points, whereas MSC treatment did not decrease this effect at week 16. There was no difference in systemic IL-6 levels between the groups. Collectively, these data show that MSC treatment does not improve the LPS-induced increase in systemic inflammation and reduction in body weight, food intake and plasma cholesterol levels.

### MSC treatment does not affect chronic LPS-induced emphysema

**Fig 4:**
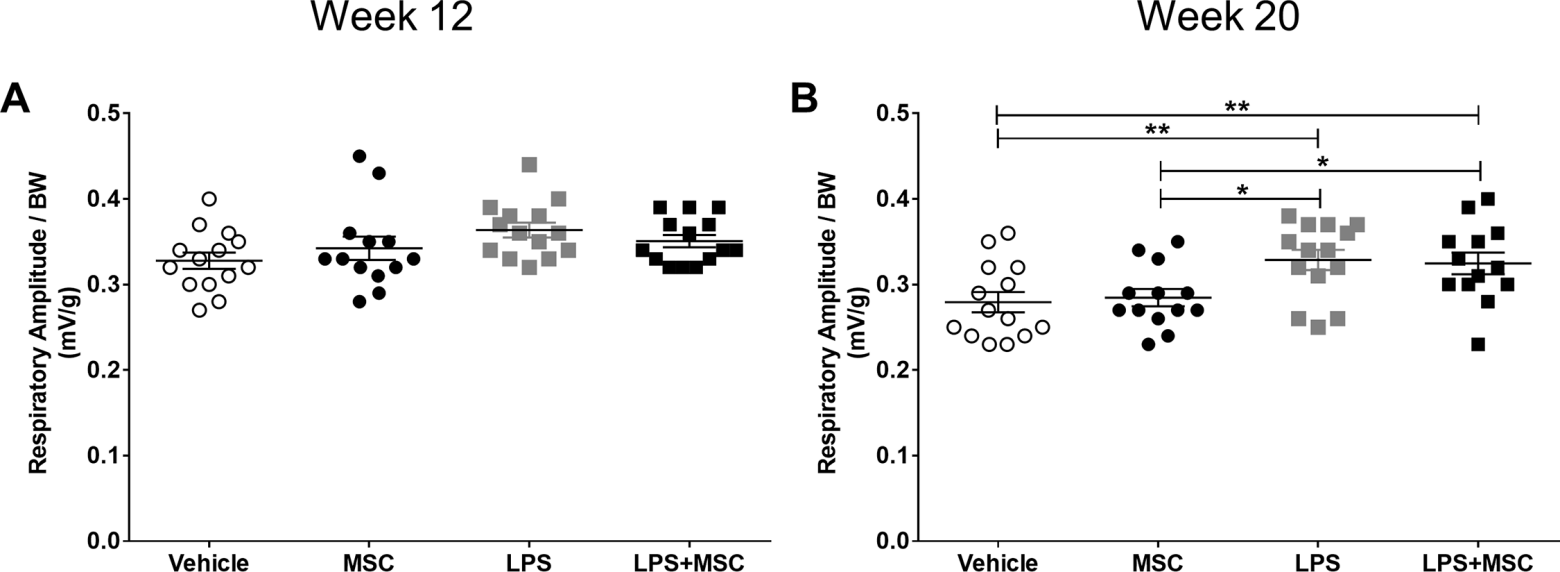
MSC treatment does not affect chronic LPS-induced decreased lung function. E3L mice were intranasally instilled with vehicle or 10 μg LPS twice weekly during 20 weeks WTD feeding. Mice received vehicle or 0.5x10^6^ MSC in week 14, 16, 18 and 20. Total respiratory amplitude was measured at 12 and 20 weeks (A and B) using non-invasive whole body phlethysmography. Data are shown as mean±SEM; n = 12–15; *p<0.05, **p<0.01.

To assess lung function, respiratory rate and peak amplitude were measured by whole body phlethysmography in week 12, just before the start of MSC treatment and at the end of the study in week 20. Respiratory rate did not differ between groups at week 12 or 20 (S5A and S5B Fig). There were no differences in peak amplitude at week 12 (Fig 4A), but peak amplitude was increased after 20 weeks of LPS instillation, indicating a lowering in lung function (Fig 4B). MSC treatment did not affect respiratory amplitude.

**Fig 5:**
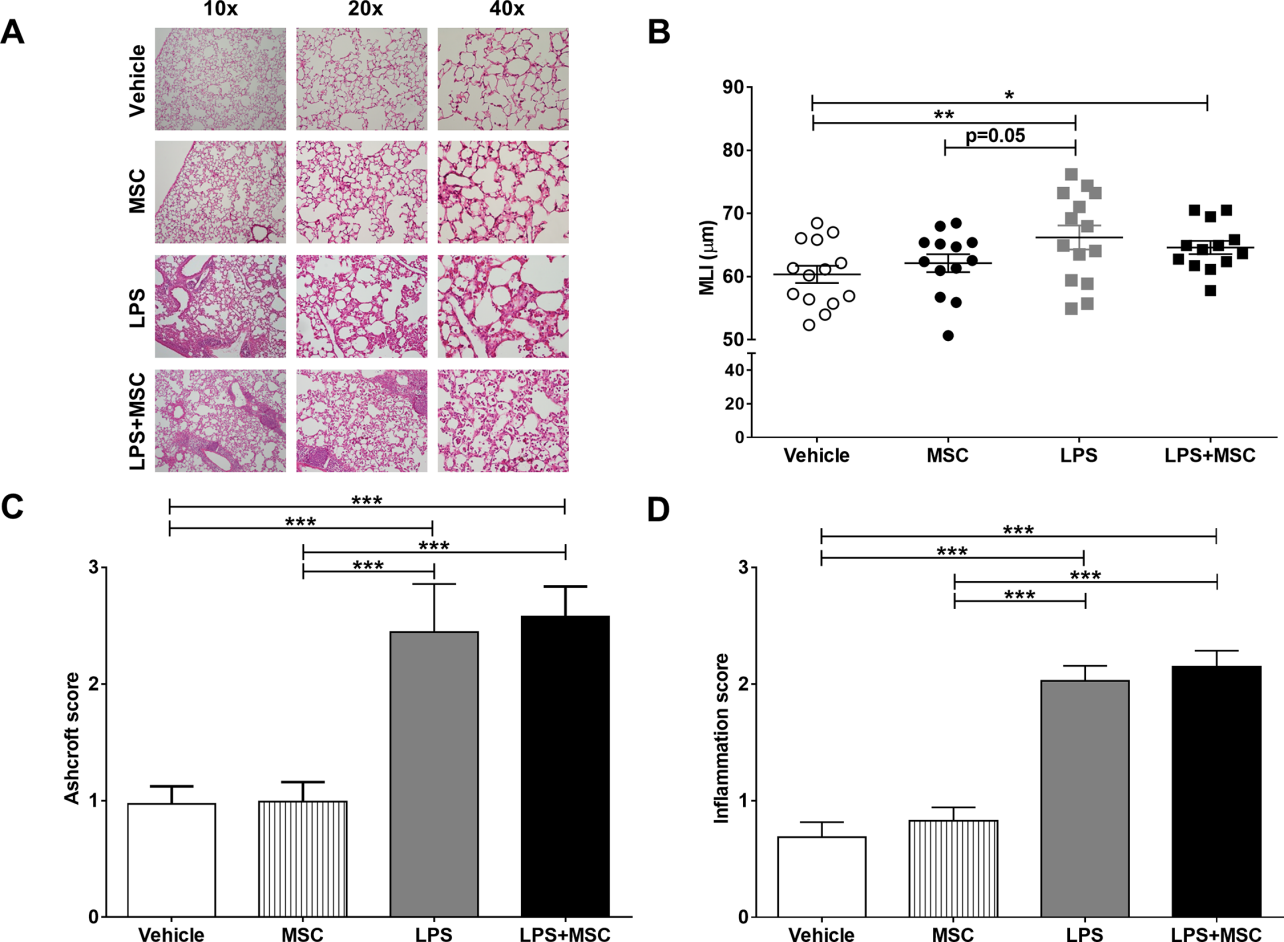
MSC treatment does not affect chronic LPS-induced emphysema, remodelling and inflammation. After sacrifice, lungs were fixed *in situ* by gentle infusion of fixative by a continuous-release pump under constant pressure (through a tracheal cannula) after which paraffin sections were prepared and stained with HE (A). Emphysema was assessed by morphometric assessment of the MLI (B), as a measurement for destruction of alveolar walls and enlargement of alveolar space. In addition, remodelling (C) and inflammatory aggregates (D) were semi-quantified on Azan-trichrome stained lung slides. Data are shown as mean±SEM; n = 12–15; *p<0.05, **p<0.01, ***p<0.001.

Emphysema was assessed by morphometric assessment of the mean linear intercept (MLI) and air/tissue (AT) ratio, indicating destruction of alveolar walls and enlargement of alveolar space. MLI was higher after chronic intranasal LPS instillation (Fig 5B) although MSC treatment did not lower MLI as a parameter for lung tissue destruction. No differences were observed in AT ratio between groups (S5C Fig), most likely explained by the high cellular influx in lung tissue that affected this measurement (Fig 5A). Furthermore, mice that were exposed to chronic intranasal LPS instillation, showed an increase in remodeling and fibrosis (Fig 5C), which was accompanied by an increased number of inflammatory aggregates (Fig 5D). These parameters were unaffected by MSC treatment. These data show that the applied MSC treatment does not reverse pulmonary inflammation, tissue remodeling and damage following chronic intranasal LPS instillation.

### MSC treatment does not affect chronic LPS-induced pulmonary inflammation

**Fig 6:**
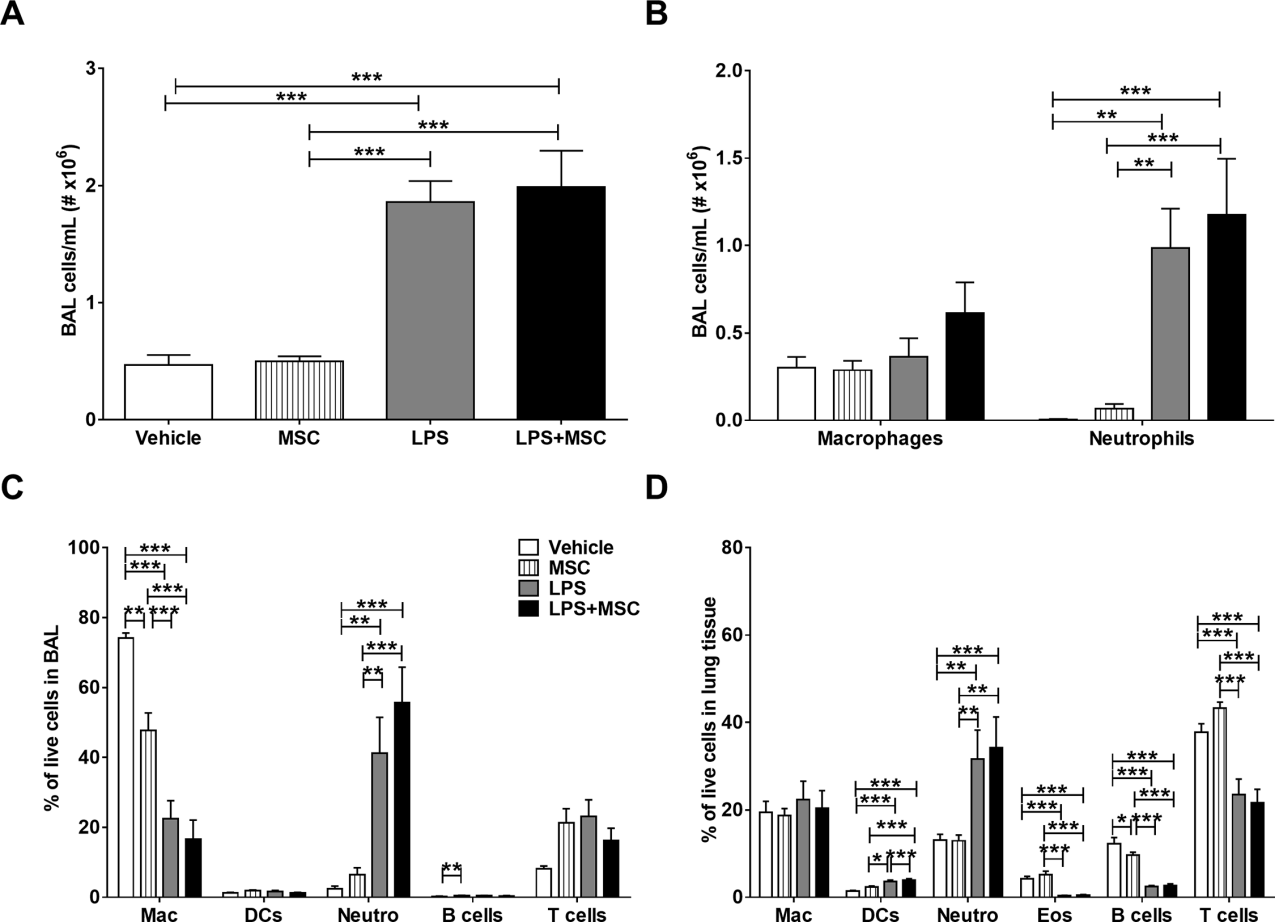
MSC treatment does not affect chronic LPS-induced pulmonary inflammation. E3L mice fed a WTD were intranasally instilled with vehicle or 10 μg LPS twice weekly during 20 weeks WTD feeding. Mice received vehicle or 0.5x10^6^ MSC in week 14, 16, 18 and 20. At sacrifice, BAL was collected for cytospin preparation and FACS analysis. Total cell number (A) and cell differentials (B) were obtained. BAL cells were also analysed using FACS analysis (C). At sacrifice, single cell suspensions were obtained from lung tissue and analysed by FACS (D). Data are shown as mean±SEM; n = 12–15;**p<0.01, ***p<0.001.

BAL fluid was collected at the end of the study and total cell counts and cell differentials were obtained using morphology and FACS analysis. LPS administration increased the total number of cells in BAL (Fig 6A), likely explained by an increased number of neutrophils (Fig 6B), which was not prevented by MSC treatment.

FACS analysis confirmed these observations, showing that LPS instillation increased the neutrophil fraction and decreased the macrophage fraction in BAL, whereas MSC treatment alone also reduced the percentage of macrophages in BAL (Fig 6C). Furthermore, in lung tissue LPS exposure decreased the percentage of B cells, T cells and eosinophils, and increased neutrophils and dendritic cells. MSC treatment had no influence on any of these cell types in lung tissue (Fig 6D). Additionally, we determined KC levels in BAL (S4A Fig), however did not find significant differences between the groups. Altogether, these data show that chronic LPS instillation increases neutrophil infiltration in the lungs, which is not inhibited upon MSC treatment.

### Chronic LPS instillation and MSC treatment do not affect atherosclerosis

**Fig 7:**
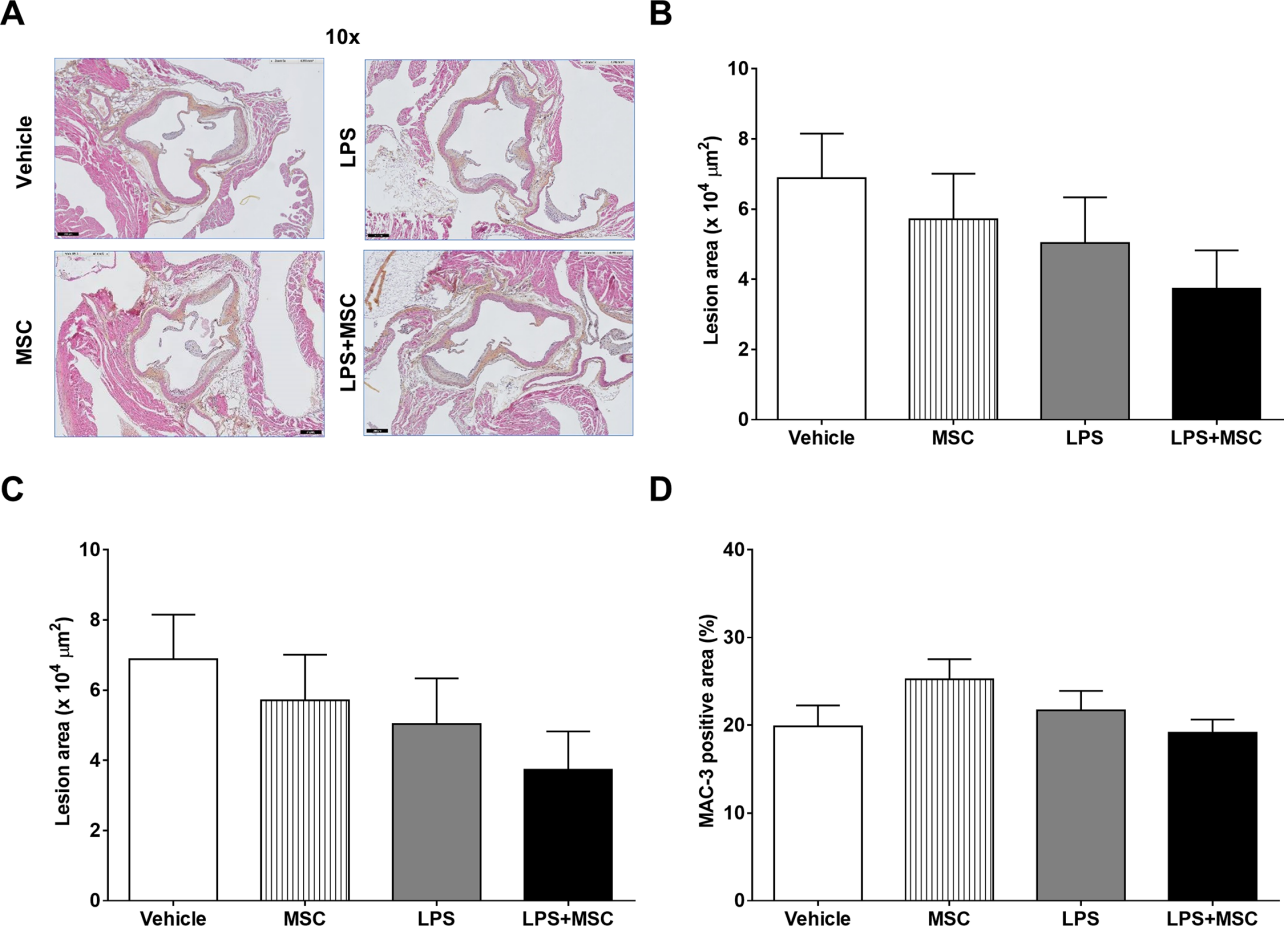
Chronic LPS instillation and MSC treatment do not affect atherosclerosis. E3L mice fed a WTD were intranasally instilled with vehicle or 10 μg LPS twice weekly during 20 weeks WTD feeding. Mice received vehicle or 0.5x10^6^ MSC in week 14, 16, 18 and 20. Hearts were isolated and fixed in phosphate-buffered 4% formaldehyde and processed for paraffin embedding. For quantification and classification of atherosclerosis, the hearts were cross-sectioned (5 μm) throughout the entire aortic root area and stained with HPS (A). Lesion area (B) and severity (C) was determined with ImageJ. MAC-3 positive macrophages content was determined (D). Data are shown as mean±SEM; n = 12–15.

Finally, we examined the effect of chronic intranasal LPS instillation and subsequent MSC treatment on atherosclerosis development. To this purpose we measured atherosclerotic lesion area and severity in the aortic root area of which representative pictures are shown in Fig 7A. There were no significant differences in atherosclerotic lesion area after LPS instillation and/or MSC treatment as shown in Fig 7B. Also, atherosclerotic lesion severity (Fig 7C), unaffected segments (S6 Fig), and MAC-3 positive macrophage content of the atherosclerotic plaques (Fig 7D) were not significantly different between the groups.

## Discussion

COPD patients have an increased risk for atherosclerosis development, as the most important underlying cause of CVD. Current therapy for COPD patients with CVD consists of standard COPD treatment with inhaled bronchodilators and anti-inflammatory agents supplemented with lipid-lowering drugs such as statins. However, the effects of COPD treatment on CVD symptoms and vice versa are not well known, and current therapy may be suboptimal. Treatment with MSC, which have been ascribed both anti-inflammatory and tissue repair-inducing properties, may represent a novel combined therapy for COPD patients with CVD. We show that MSC treatment inhibits pulmonary and systemic inflammation in acute LPS-induced pulmonary inflammation in atherosclerosis-prone *E3L* mice. Whereas MSC treatment did show promising results in the acute study, chronic MSC treatment did not affect pulmonary inflammation, emphysema or atherosclerosis development.

To determine the effect of MSC treatment in an acute study, we intranasally instilled *E3L* mice with LPS twice, which induced neutrophil infiltration in BAL fluid and an increase in systemic IL-6 levels. The pulmonary neutrophilic as well as the systemic IL-6 response to LPS instillation were inhibited by intravenous MSC injection. These data are in line with several other studies that showed a dampened inflammatory response upon MSC treatment. For example, intrapleural MSC administration inhibited lung injury and inflammation in rats after endotoxin-induced acute lung injury. Furthermore, inflammatory cytokines such as TNF-α and BAL fluid cell count were reduced upon MSC treatment, but this was not accompanied by a reduction in neutrophils [14]. Intravenous MSC administration also improved survival after *Pseudomonas aeruginosa*-induced peritoneal sepsis, which was most likely due to an increase in bacterial phagocytosis by macrophages [33]. Hoogduijn et al. [34] showed that intravenous injected MSC home to the lung, where they induce an inflammatory response as shown by increased TNF-α, MCP-1 and IL-1β mRNA levels. Also, systemic cytokines such as IL-6 and TNF-α were increased upon MSC injection. However, MSC injection 3 days prior to intravenous LPS injection did dampen the LPS-induced systemic inflammatory response. This suggests that MSC injection induces an early inflammatory response, which may reduce subsequent inflammatory responses [35]. Other published studies show beneficial effects of MSC treatment on emphysema. Intratracheal or intravenous (through the jugular vein) administration of MSC isolated from either bone marrow, lung or adipose tissue lowered porcine pancreas elastase (PPE)-induced pulmonary damage and (acute) inflammation, most likely through inhibition of pro-inflammatory macrophages, induction of anti-inflammatory macrophages and production of angiogenic factors [36–38]. Also, papain- [19, 20] and cigarette smoke-induced [16, 18, 39–41] pulmonary tissue destruction and emphysema development were reduced upon MSC treatment. In the chronic study we assessed the effect of MSC treatment on LPS-induced pulmonary inflammation, emphysema and atherosclerosis development in *E3L* mice. In the current study, we used a high intranasal LPS dose, to induce COPD-like features, including persistent airway inflammation and emphysema [42, 43]. Although the MLI in LPS-exposed animals was increased, the extent of alveolar tissue damage was limited compared to for example porcine pancreatic elastase (PPE)-induced emphysema [28]. However, there was an increase in cellular infiltration in the lung and BAL after repeated intranasal LPS instillation. In conclusion, our data confirm the effect of MSC treatment on lung and systemic inflammation in our acute model, but do not confirm the effect on emphysema development in a chronic LPS model.

We previously showed that low-dose intranasal LPS administration caused a limited, but significant increase in the atherosclerotic lesion area [28]. In the current study, we used a higher LPS dosage. When studying atherosclerosis development in the chronic model, we observed that repeated intranasal LPS administration during 20 weeks induced a lowering in body weight, most likely due to a reduction in food intake. A reduction in food intake results in a reduced cholesterol intake, which thus explains the decreased cholesterol exposure in LPS-exposed mice. In this study, we used *E3L* mice, which upon feeding of a Western-type diet containing cholesterol, develop diet-induced atherosclerosis. Plasma cholesterol levels and atherosclerotic lesion area are strongly correlated [44], which may explain the lack of increased atherosclerosis development after intranasal LPS administration. The reduced food intake and resulting lower cholesterol exposure may have outweighed a potential effect of LPS-induced systemic inflammation on atherosclerosis development.

In the chronic study mice were treated with MSC from week 14 onwards as therapeutic treatment, rather than a prophylactic treatment. MSC treatment in the chronic study, did not affect LPS-induced inflammation in the lung. One of the likely explanations may be the limited number of therapeutic treatments relative to the duration and number of LPS instillations. Whereas most studies described above have established emphysema and administer the MSC after stimulation with for example cigarette smoke [20, 39], our chronic study mimics MSC treatment during ongoing chronic disease with repeated LPS administration rather that patients who have ceased smoking. A recent review describes two polarized phenotypes of MSC, which are either pro- or anti-inflammatory [45]. Chronic intranasal LPS administration may induce leakage of LPS, which can induce development of a pro-inflammatory phenotype of the infused MSC, explaining the lack of immunomodulation in our chronic model. Another explanation for the lack of effect on inflammation and emphysema development in the chronic model is the use of hyperlipidemic recipient mice, in contrast to other studies using normolipidemic recipients. Xu *et al*. [46] showed that hyperlipidemia may compromise homing efficiency of systemically administered MSC and inhibits bone regeneration. Although this is a different disease model, hyperlipidemia may have also influenced MSC trafficking in our chronic model, which could explain that MSC treatment did not lower atherosclerosis and emphysema development in the current study. Furthermore, a study by Frodermann *et al. [26]*, where MSC were adoptively transferred in *LDLr-/-* mice, showed lowered cholesterol levels and atherosclerosis development. Whereas MSC were isolated from WT mice in that study, we used MSC isolated from *E3L* mice, which may be functionally different from WT MSC, however future research is necessary to determine this. And whereas some studies show that MSC treatment can stabilize and repair ruptured plaques and may contribute to treatment of CVD [25, 47], there is also evidence that MSC treatment may induce vascular remodeling and calcification [48, 49].

In conclusion, although in our chronic study we did not find an effect of MSC treatment on pulmonary inflammation, emphysema and atherosclerosis development, in our acute study we show that MSC inhibits both the intranasal LPS-induced cellular influx into the lung as well as the systemic inflammatory response, and may thus be further explored as a strategy for combined treatment of COPD and CVD.

## Supporting information

S1 Fig Study outline and baseline parameters in the acute study. The effect and feasibility of MSC treatment on LPS-induced acute pulmonary and systemic inflammation was determined in the acute study as outlined in (A). Mice were matched at baseline based on bodyweight (B) and plasma lipids (C). Ten μg LPS (serotype 055:B5 *Escherichia coli* LPS) in 50 μl sterile PBS was administered intranasally twice (*i*.*e*. on day 1 and 3). Control mice received 50 μl sterile PBS (vehicle). MSC (0.5x106 cells in 200 μl PBS) or 200 μl sterile PBS as control was administered intravenously by tail vein injection on day 1 and 2. Blood was collected after LPS administration to determine the systemic IL-6 response. Mice were sacrificed on day 4.
(TIF)

S2 Fig Study outline and baseline parameters in the chronic study. In the *chronic* study the effect of MSC treatment on LPS-induced chronic pulmonary and systemic inflammation, emphysema and atherosclerosis development was determined as outlined in (A). Mice were matched at baseline based on bodyweight (B) and plasma lipids (C). Ten μg LPS in 50 μl sterile PBS was intranasally administered twice weekly, during 20 weeks WTD feeding to induce diet-induced atherosclerosis. Control mice received 50 μl sterile PBS (vehicle). From week 14 onwards, mice received MSC intravenously by tail vein injection every other week (*i*.*e*. week 14, 16, 18 and 20). Blood was collected every 4 weeks to determine plasma lipid levels. Mice were sacrificed 24 h after the last LPS instillation.
(TIF)

S3 Fig Characteristics and *in vitro* anti-inflammatory actions of isolated MSC. MSC were isolated from bone marrow of female donor *E3L* mice and MSC of passage 4–7 were used throughout the study. MSC were characterized based on the presence and absence of classical markers (A) and the effect on T cell proliferation was determined *in vitro* (B). Data in B are shown as mean±SEM.
(TIF)

S4 Fig LPS exposure or MSC treatment do not affect BAL KC levels or systemic IL-6 levels. (A) Levels of KC were measured in BAL at the end of the study. (B) Levels of IL-6 were measured in plasma of mice at week 11 and week 16 (before and after MSC treatment). Data for A and B are shown as mean±SEM; n = 12–15.
(TIF)

S5 Fig LPS and MSC treatment do not affect respiration rate and AT- ratio. E3L mice were intranasally instilled with vehicle or 10 μg LPS twice weekly during 20 weeks WTD feeding. Mice received vehicle or 0.5x10^6^ MSC in week 14, 16, 18 and 20. Respiratory rate was measured at 12 and 20 weeks (A and B) using non-invasive whole body phlethysmography. Air-tissue ratio was assessed by morphometric assessment (C). Data are shown as mean±SEM; n = 12–15.
(TIF)

S6 Fig Chronic LPS instillation and MSC treatment do not affect undiseased atherosclerotic segments. E3L mice fed a WTD were intranasally instilled with vehicle or 10 μg LPS twice weekly during 20 weeks WTD feeding. Mice received vehicle or 0.5x10^6^ MSC in week 14, 16, 18 and 20. Hearts were isolated and fixed in phosphate-buffered 4% formaldehyde and processed for paraffin embedding. Numbers of unaffected segments were determined. Data are shown as mean±SEM; n = 12–15.
(TIF)

## Abbreviations

AT: air-tissue
BAL: bronchoalveolar lavage
COPD: chronic obstructive pulmonary disease
CRP: C-reactive protein
CVD: cardiovascular disease
E3L: *APOE***3-Leiden*
HE: haematoxilin-eosin
HPS: haematoxilin-phloxin-saffron
IL: interleukin
LPS: lipopolysaccharide
MLI: mean linear intercept
MSC: mesenchymal stromal cell
PL: phospholipids
PPE: porcine pancreatic elastase
RT: room temperature
SAA: serum amyloid A
TC: total cholesterol
TG: triglycerides
TNF-α: tumor necrosis factor- α
WTD: Western-type diet

## References

[1] From the Global Strategy for the Diagnosis, Management and Prevention of COPD, Global Initiative for Chronic Obstructive Lung Disease (GOLD) 2016. Available from: http://www.goldcopd.org/. 2016. 10.3760/cma.j.issn.0376-2491.2016.34.001 27667101

[2] Vivodtzev I, Tamisier R, Baguet JP, Borel JC, Levy P, Pepin JL. Arterial stiffness in COPD. Chest. 2014;145(4):861–75. doi: 1852921 [pii]; doi: 10.1378/chest.13-1809 24687708 10.1378/chest.13-1809

[3] Nussbaumer-Ochsner Y, Rabe KF. Systemic manifestations of COPD. Chest. 2011;139(1):165–73. doi: 139/1/165 [pii]; doi: 10.1378/chest.10-1252 21208876 10.1378/chest.10-1252

[4] Libby P, Hansson GK. Inflammation and immunity in diseases of the arterial tree: players and layers. Circ Res. 2015;116(2):307–11. doi: CIRCRESAHA.116.301313 [pii]; doi: 10.1161/CIRCRESAHA.116.301313 25593275 10.1161/CIRCRESAHA.116.301313 PMC4299915

[5] Barua RS, Sharma M, Dileepan KN. Cigarette Smoke Amplifies Inflammatory Response and Atherosclerosis Progression Through Activation of the H1R-TLR2/4-COX2 Axis. Front Immunol. 2015;6:572 doi: 10.3389/fimmu.2015.00572 ; PubMed Central PMCID: PMCPMC4638143. 26617606 10.3389/fimmu.2015.00572 PMC4638143

[6] Fabbri LM, Luppi F, Beghe B, Rabe KF. Complex chronic comorbidities of COPD. Eur Respir J. 2008;31(1):204–12. doi: 31/1/204 [pii]; doi: 10.1183/09031936.00114307 18166598 10.1183/09031936.00114307

[7] Fabbri LM, Rabe KF. From COPD to chronic systemic inflammatory syndrome? Lancet. 2007;370(9589):797–9. doi: S0140-6736(07)61383-X [pii]; doi: 10.1016/S0140-6736(07)61383-X 17765529 10.1016/S0140-6736(07)61383-X

[8] Luppi F, Franco F, Beghe B, Fabbri LM. Treatment of chronic obstructive pulmonary disease and its comorbidities. Proc Am Thorac Soc. 2008;5(8):848–56. doi: 5/8/848 [pii]; doi: 10.1513/pats.200809-101TH 19017740 10.1513/pats.200809-101TH

[9] Fruchter O, Yigla M, Kramer MR. Lipid profile and statin use: the paradox of survival after acute exacerbation of chronic obstructive pulmonary disease. Am J Med Sci. 2015;349(4):338–43. doi: 10.1097/MAJ.0000000000000435 25719977 10.1097/MAJ.0000000000000435

[10] Schepers K, Fibbe WE. Unraveling mechanisms of mesenchymal stromal cell-mediated immunomodulation through patient monitoring and product characterization. Ann N Y Acad Sci. 2016;1370(1):15–23. doi: 10.1111/nyas.12984 . 26713608 10.1111/nyas.12984

[11] Bernardo ME, Fibbe WE. Mesenchymal stromal cells and hematopoietic stem cell transplantation. Immunol Lett. 2015;168(2):215–21. doi: 10.1016/j.imlet.2015.06.013 . 26116911 10.1016/j.imlet.2015.06.013

[12] Eggenhofer E, Benseler V, Kroemer A, Popp FC, Geissler EK, Schlitt HJ, et al Mesenchymal stem cells are short-lived and do not migrate beyond the lungs after intravenous infusion. Front Immunol. 2012;3:297 doi: 10.3389/fimmu.2012.00297 23056000 10.3389/fimmu.2012.00297 PMC3458305

[13] Duijvestein M, Wildenberg ME, Welling MM, Hennink S, Molendijk I, van Zuylen VL, et al Pretreatment with interferon-gamma enhances the therapeutic activity of mesenchymal stromal cells in animal models of colitis. Stem Cells. 2011;29(10):1549–58. doi: 10.1002/stem.698 21898680 10.1002/stem.698

[14] Qin ZH, Xu JF, Qu JM, Zhang J, Sai Y, Chen CM, et al Intrapleural delivery of MSCs attenuates acute lung injury by paracrine/endocrine mechanism. J Cell Mol Med. 2012;16(11):2745–53. doi: 10.1111/j.1582-4934.2012.01597.x 22697354 10.1111/j.1582-4934.2012.01597.x PMC4118243

[15] Fibbe WE, Bernardo ME. Control of immune responses by mesenchymal stromal cells. Rinsho Ketsueki. 2014;55(10):2190–4. doi: DN/JST.JSTAGE/rinketsu/55.2190 [pii]. 25297786

[16] Gu W, Song L, Li XM, Wang D, Guo XJ, Xu WG. Mesenchymal stem cells alleviate airway inflammation and emphysema in COPD through down-regulation of cyclooxygenase-2 via p38 and ERK MAPK pathways. Sci Rep. 2015;5:8733. doi: srep08733 [pii]; doi: 10.1038/srep08733 25736434 10.1038/srep08733 PMC4348625

[17] Gupta N, Krasnodembskaya A, Kapetanaki M, Mouded M, Tan X, Serikov V, et al Mesenchymal stem cells enhance survival and bacterial clearance in murine Escherichia coli pneumonia. Thorax. 2012;67(6):533–9. doi: thoraxjnl-2011-201176 [pii]; doi: 10.1136/thoraxjnl-2011-201176 22250097 10.1136/thoraxjnl-2011-201176 PMC3358432

[18] Huh JW, Kim SY, Lee JH, Lee JS, Van TQ, Kim M, et al Bone marrow cells repair cigarette smoke-induced emphysema in rats. Am J Physiol Lung Cell Mol Physiol. 2011;301(3):L255–L66. doi: ajplung.00253.2010 [pii]; doi: 10.1152/ajplung.00253.2010 21622846 10.1152/ajplung.00253.2010

[19] Zhen G, Xue Z, Zhao J, Gu N, Tang Z, Xu Y, et al Mesenchymal stem cell transplantation increases expression of vascular endothelial growth factor in papain-induced emphysematous lungs and inhibits apoptosis of lung cells. Cytotherapy. 2010;12(5):605–14. doi: 10.3109/14653241003745888 20429787 10.3109/14653241003745888

[20] Zhen G, Liu H, Gu N, Zhang H, Xu Y, Zhang Z. Mesenchymal stem cells transplantation protects against rat pulmonary emphysema. Front Biosci. 2008;13:3415–22. doi: 2936 [pii]. 18508443 10.2741/2936

[21] Stolk J, Broekman W, Mauad T, Zwaginga JJ, Roelofs H, Fibbe WE, et al A phase I study for intravenous autologous mesenchymal stromal cell administration to patients with severe emphysema. QJM. 2016;109(5):331–6. doi: 10.1093/qjmed/hcw001 ; PubMed Central PMCID: PMCPMC4888332. 26819296 10.1093/qjmed/hcw001 PMC4888332

[22] Weiss DJ. Concise review: current status of stem cells and regenerative medicine in lung biology and diseases. Stem Cells. 2014;32(1):16–25. doi: 10.1002/stem.1506 23959715 10.1002/stem.1506 PMC4208500

[23] Weiss DJ, Casaburi R, Flannery R, LeRoux-Williams M, Tashkin DP. A placebo-controlled, randomized trial of mesenchymal stem cells in COPD. Chest. 2013;143(6):1590–8. doi: 10.1378/chest.12-2094 ; PubMed Central PMCID: PMCPMC4694112. 23172272 10.1378/chest.12-2094 PMC4694112

[24] Atsma DE, Fibbe WE, Rabelink TJ. Opportunities and challenges for mesenchymal stem cell-mediated heart repair. Curr Opin Lipidol. 2007;18(6):645–9. doi: 10.1097/MOL.0b013e3282f0dd1f 00041433-200712000-00007 [pii]. 17993810 10.1097/MOL.0b013e3282f0dd1f

[25] Fang SM, Du DY, Li YT, Ge XL, Qin PT, Zhang QH, et al Allogeneic bone marrow mesenchymal stem cells transplantation for stabilizing and repairing of atherosclerotic ruptured plaque. Thromb Res. 2013;131(6):e253–e7. doi: S0049-3848(13)00134-5 [pii]; doi: 10.1016/j.thromres.2013.04.002 23618388 10.1016/j.thromres.2013.04.002

[26] Frodermann V, van DJ, van PM, van Santbrink PJ, Bot I, Kuiper J, et al Mesenchymal Stem Cells Reduce Murine Atherosclerosis Development. Sci Rep. 2015;5:15559. doi: srep15559 [pii]; doi: 10.1038/srep15559 26490642 10.1038/srep15559 PMC4614841

[27] van Vlijmen BJ, van den Maagdenberg AM, Gijbels MJ, van der Boom H, HogenEsch H, Frants RR, et al Diet-induced hyperlipoproteinemia and atherosclerosis in apolipoprotein E3-Leiden transgenic mice. J Clin Invest. 1994;93(4):1403–10. doi: 10.1172/JCI117117 8163645 10.1172/JCI117117 PMC294153

[28] Khedoe PP, Wong MC, Wagenaar GT, Plomp JJ, Van EM, Havekes LM, et al The effect of PPE-induced emphysema and chronic LPS-induced pulmonary inflammation on atherosclerosis development in APOE*3-LEIDEN mice. PLoS One. 2013;8(11):e80196 doi: 10.1371/journal.pone.0080196 PONE-D-13-27131 [pii]. 24303000 10.1371/journal.pone.0080196 PMC3841138

[29] Soleimani M, Nadri S. A protocol for isolation and culture of mesenchymal stem cells from mouse bone marrow. Nat Protoc. 2009;4(1):102–6. doi: nprot.2008.221 [pii]; doi: 10.1038/nprot.2008.221 19131962 10.1038/nprot.2008.221

[30] Ashcroft T, Simpson JM, Timbrell V. Simple method of estimating severity of pulmonary fibrosis on a numerical scale. J Clin Pathol. 1988;41(4):467–70. ; PubMed Central PMCID: PMCPMC1141479. 3366935 10.1136/jcp.41.4.467 PMC1141479

[31] Bayes HK, Ritchie N, Irvine S, Evans TJ. A murine model of early Pseudomonas aeruginosa lung disease with transition to chronic infection. Sci Rep. 2016;6:35838 doi: 10.1038/srep35838 ; PubMed Central PMCID: PMCPMC5090221. 27804985 10.1038/srep35838 PMC5090221

[32] Zadelaar AS, Boesten LS, Jukema JW, van Vlijmen BJ, Kooistra T, Emeis JJ, et al Dual PPARalpha/gamma agonist tesaglitazar reduces atherosclerosis in insulin-resistant and hypercholesterolemic ApoE*3Leiden mice. Arterioscler Thromb Vasc Biol. 2006;26(11):2560–6. doi: 01.ATV.0000242904.34700.66 [pii]; doi: 10.1161/01.ATV.0000242904.34700.66 16931788 10.1161/01.ATV.0000242904.34700.66

[33] Krasnodembskaya A, Samarani G, Song Y, Zhuo H, Su X, Lee JW, et al Human mesenchymal stem cells reduce mortality and bacteremia in gram-negative sepsis in mice in part by enhancing the phagocytic activity of blood monocytes. Am J Physiol Lung Cell Mol Physiol. 2012;302(10):L1003–L13. doi: ajplung.00180.2011 [pii]; doi: 10.1152/ajplung.00180.2011 22427530 10.1152/ajplung.00180.2011 PMC3362255

[34] Hoogduijn MJ, Roemeling-van RM, Engela AU, Korevaar SS, Mensah FK, Franquesa M, et al Mesenchymal stem cells induce an inflammatory response after intravenous infusion. Stem Cells Dev. 2013;22(21):2825–35. doi: 10.1089/scd.2013.0193 23767885 10.1089/scd.2013.0193

[35] Eggenhofer E, Luk F, Dahlke MH, Hoogduijn MJ. The life and fate of mesenchymal stem cells. Front Immunol. 2014;5:148 doi: 10.3389/fimmu.2014.00148 24904568 10.3389/fimmu.2014.00148 PMC4032901

[36] Antunes MA, Abreu SC, Cruz FF, Teixeira AC, Lopes-Pacheco M, Bandeira E, et al Effects of different mesenchymal stromal cell sources and delivery routes in experimental emphysema. Respir Res. 2014;15:118. doi: s12931-014-0118-x [pii]; doi: 10.1186/s12931-014-0118-x 25272959 10.1186/s12931-014-0118-x PMC4189723

[37] Tibboel J, Keijzer R, Reiss I, de Jongste JC, Post M. Intravenous and intratracheal mesenchymal stromal cell injection in a mouse model of pulmonary emphysema. COPD. 2014;11(3):310–8. doi: 10.3109/15412555.2013.854322 24295402 10.3109/15412555.2013.854322 PMC4046870

[38] Katsha AM, Ohkouchi S, Xin H, Kanehira M, Sun R, Nukiwa T, et al Paracrine factors of multipotent stromal cells ameliorate lung injury in an elastase-induced emphysema model. Mol Ther. 2011;19(1):196–203. doi: mt2010192 [pii]; doi: 10.1038/mt.2010.192 20842104 10.1038/mt.2010.192 PMC3017437

[39] Guan XJ, Song L, Han FF, Cui ZL, Chen X, Guo XJ, et al Mesenchymal stem cells protect cigarette smoke-damaged lung and pulmonary function partly via VEGF-VEGF receptors. J Cell Biochem. 2013;114(2):323–35. doi: 10.1002/jcb.24377 22949406 10.1002/jcb.24377

[40] Schweitzer KS, Johnstone BH, Garrison J, Rush NI, Cooper S, Traktuev DO, et al Adipose stem cell treatment in mice attenuates lung and systemic injury induced by cigarette smoking. Am J Respir Crit Care Med. 2011;183(2):215–25. doi: 201001-0126OC [pii]; doi: 10.1164/rccm.201001-0126OC 20709815 10.1164/rccm.201001-0126OC PMC3040390

[41] Song L, Guan XJ, Chen X, Cui ZL, Han FF, Guo XJ, et al Mesenchymal stem cells reduce cigarette smoke-induced inflammation and airflow obstruction in rats via TGF-beta1 signaling. COPD. 2014;11(5):582–90. doi: 10.3109/15412555.2014.898032 24766333 10.3109/15412555.2014.898032

[42] Ojo O, Lagan AL, Rajendran V, Spanjer A, Chen L, Sohal SS, et al Pathological changes in the COPD lung mesenchyme—novel lessons learned from in vitro and in vivo studies. Pulm Pharmacol Ther. 2014;29(2):121–8. doi: S1094-5539(14)00042-X [pii]; doi: 10.1016/j.pupt.2014.04.004 24747433 10.1016/j.pupt.2014.04.004

[43] Pera T, Zuidhof AB, Smit M, Menzen MH, Klein T, Flik G, et al Arginase inhibition prevents inflammation and remodeling in a guinea pig model of chronic obstructive pulmonary disease. J Pharmacol Exp Ther. 2014;349(2):229–38. doi: 10.1124/jpet.113.210138 . 24563530 10.1124/jpet.113.210138

[44] Havekes LM, van Vlijmen BJ, Jong MC, van Dijk KW, Hofker MH. Use of transgenic mice in lipoprotein metabolism and atherosclerosis research. Prostaglandins Leukot Essent Fatty Acids. 1997;57(4–5):463–6. doi: S0952-3278(97)90429-4 [pii]. 9430397 10.1016/s0952-3278(97)90429-4

[45] Bernardo ME, Fibbe WE. Mesenchymal stromal cells: sensors and switchers of inflammation. Cell Stem Cell. 2013;13(4):392–402. doi: S1934-5909(13)00406-2 [pii]; doi: 10.1016/j.stem.2013.09.006 24094322 10.1016/j.stem.2013.09.006

[46] Xu QC, Hao PJ, Yu XB, Chen SL, Yu MJ, Zhang J, et al Hyperlipidemia compromises homing efficiency of systemically transplanted BMSCs and inhibits bone regeneration. Int J Clin Exp Pathol. 2014;7(4):1580–7. 24817954 PMC4014238

[47] Abedin M, Tintut Y, Demer LL. Mesenchymal stem cells and the artery wall. Circ Res. 2004;95(7):671–6. doi: 10.1161/01.RES.0000143421.27684.12 95/7/671 [pii]. 15459088 10.1161/01.RES.0000143421.27684.12

[48] Li M, Li S, Yu L, Wu J, She T, Gan Y, et al Bone mesenchymal stem cells contributed to the neointimal formation after arterial injury. PLoS One. 2013;8(12):e82743 doi: 10.1371/journal.pone.0082743 PONE-D-13-34523 [pii]. 24349351 10.1371/journal.pone.0082743 PMC3857273

[49] Liao J, Chen X, Li Y, Ge Z, Duan H, Zou Y, et al Transfer of bone-marrow-derived mesenchymal stem cells influences vascular remodeling and calcification after balloon injury in hyperlipidemic rats. J Biomed Biotechnol. 2012;2012:165296 doi: 10.1155/2012/165296 22665980 10.1155/2012/165296 PMC3361346

