# Supplementary figures and images for: Acute and chronic effects of treatment with mesenchymal stromal cells on LPS-induced pulmonary inflammation, emphysema and atherosclerosis development
Source: PLoS One. 2017 Sep 14;12(9):e0183741. doi: 10.1371/journal.pone.0183741 (PMC5598950; doi:10.1371/journal.pone.0183741)

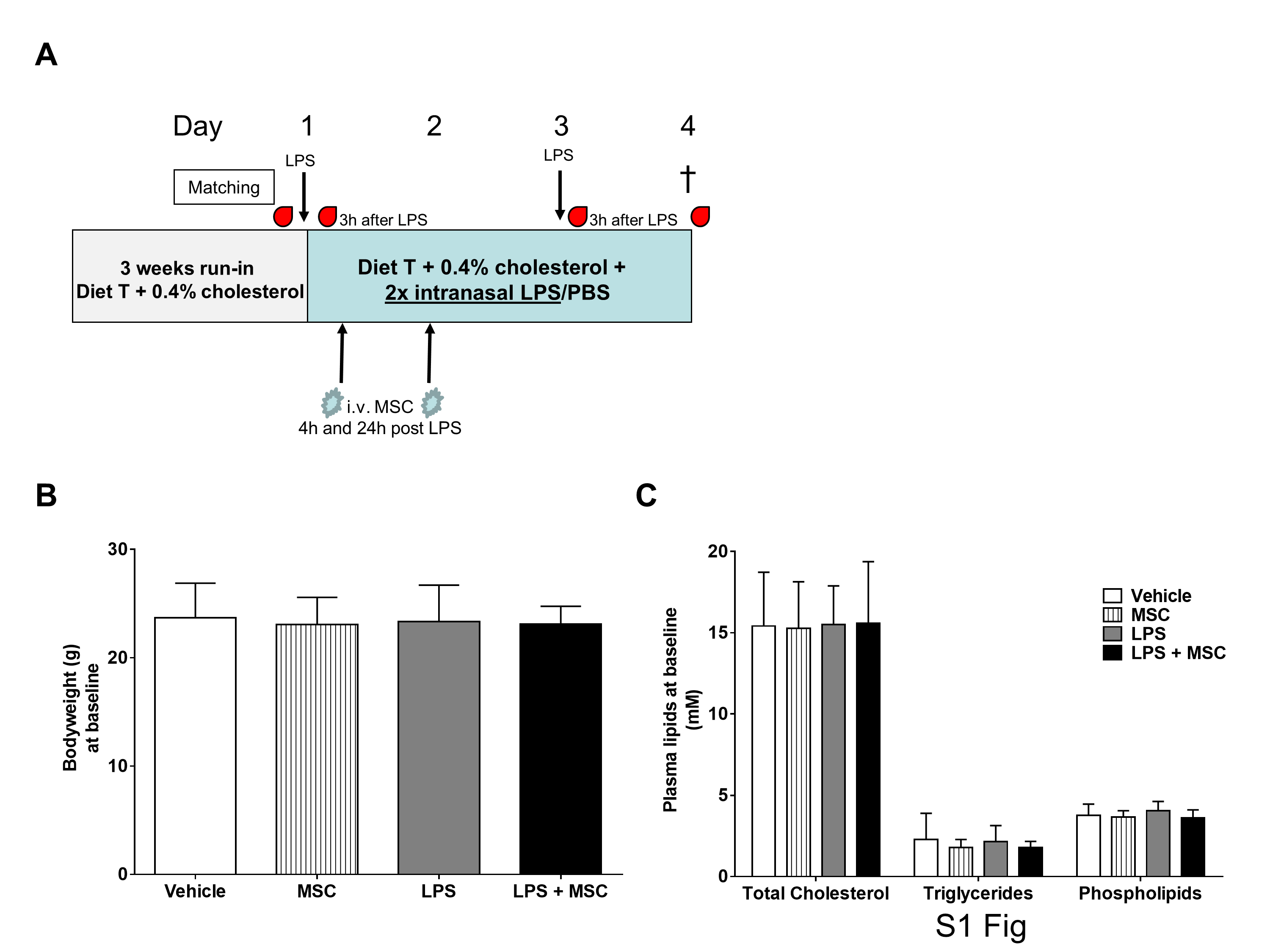

Supplement: S1 Fig — The effect and feasibility of MSC treatment on LPS-induced acute pulmonary and systemic inflammation was determined in the acute study as outlined in (A). Mice were matched at baseline based on bodyweight (B) and plasma lipids (C). Ten μg LPS (serotype 055:B5 Escherichia coli LPS) in 50 μl sterile PBS was administered intranasally twice (i.e. on day 1 and 3). Control mice received 50 μl sterile PBS (vehicle). MSC (0.5x10^6^ cells in 200 μl PBS) or 200 μl sterile PBS as control was administered intravenously by tail vein injection on day 1 and 2. Blood was collected after LPS administration to determine the systemic IL-6 response. Mice were sacrificed on day 4. (TIF) [file pone.0183741.s001.tif]

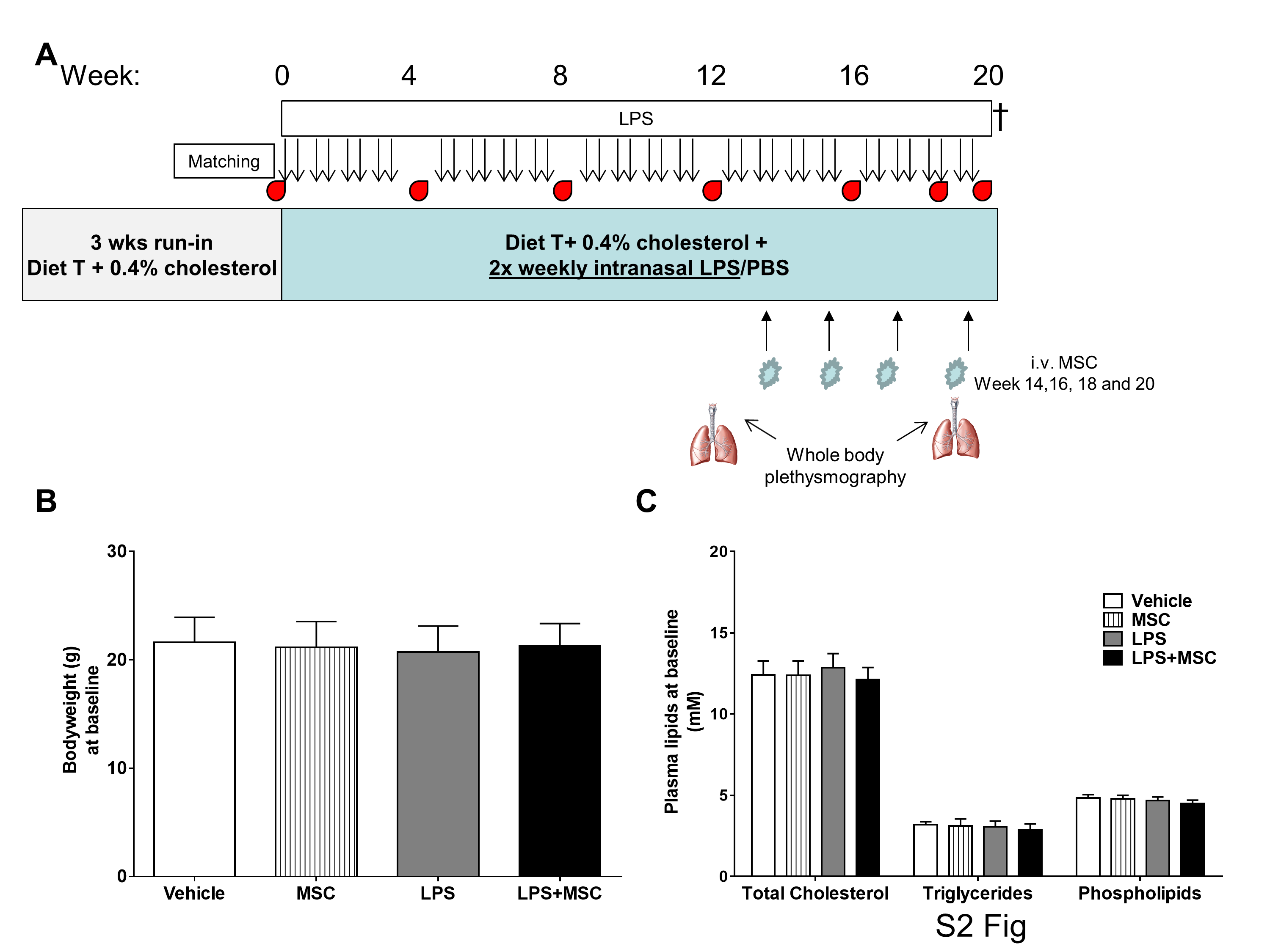

Supplement: S2 Fig — In the chronic study the effect of MSC treatment on LPS-induced chronic pulmonary and systemic inflammation, emphysema and atherosclerosis development was determined as outlined in (A). Mice were matched at baseline based on bodyweight (B) and plasma lipids (C). Ten μg LPS in 50 μl sterile PBS was intranasally administered twice weekly, during 20 weeks WTD feeding to induce diet-induced atherosclerosis. Control mice received 50 μl sterile PBS (vehicle). From week 14 onwards, mice received MSC intravenously by tail vein injection every other week (i.e. week 14, 16, 18 and 20). Blood was collected every 4 weeks to determine plasma lipid levels. Mice were sacrificed 24 h after the last LPS instillation. (TIF) [file pone.0183741.s002.tif]

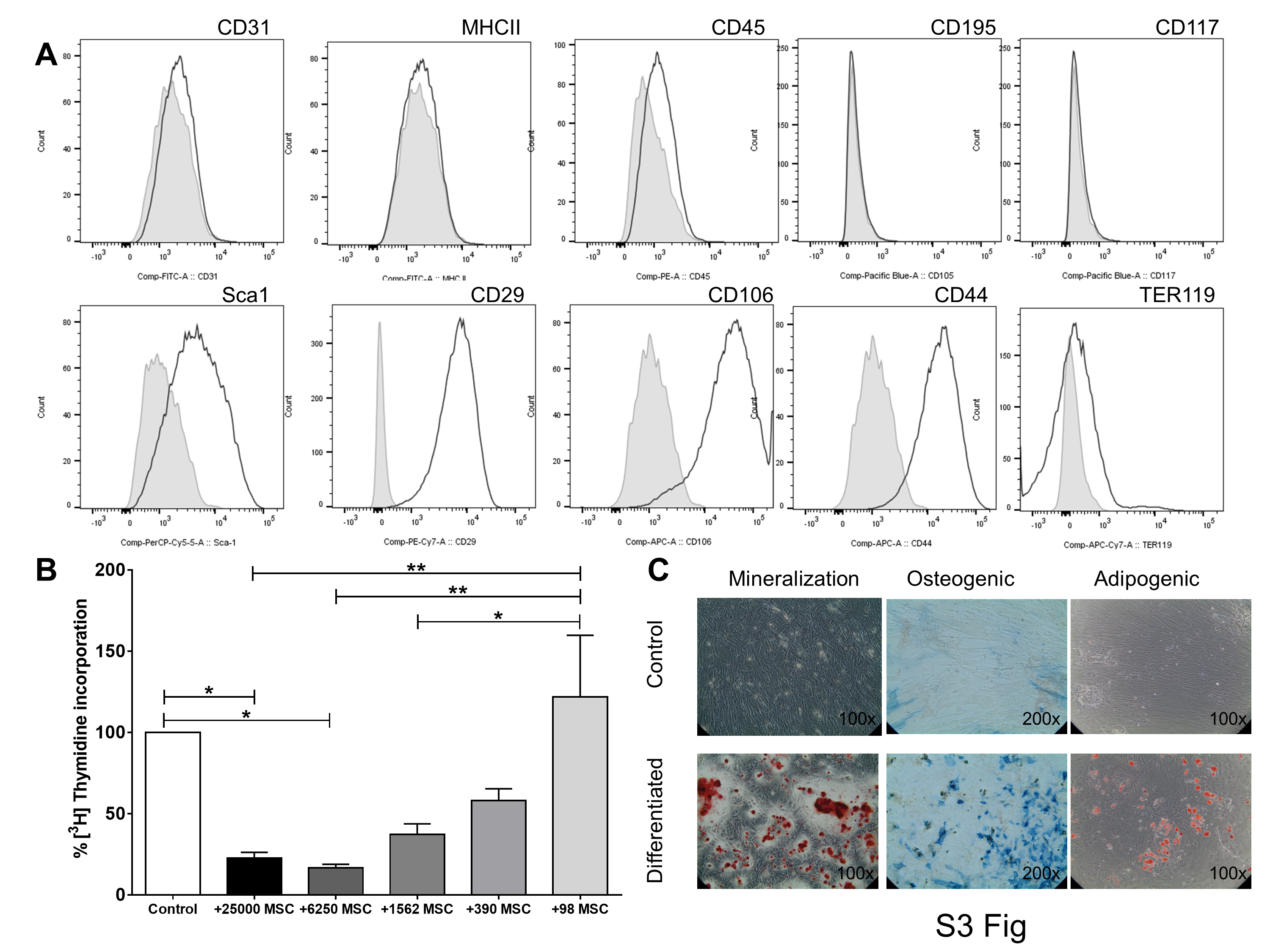

Supplement: S3 Fig — MSC were isolated from bone marrow of female donor E3L mice and MSC of passage 4–7 were used throughout the study. MSC were characterized based on the presence and absence of classical markers (A) and the effect on T cell proliferation was determined in vitro (B). Data in B are shown as mean±SEM. (TIF) [file pone.0183741.s003.tif]

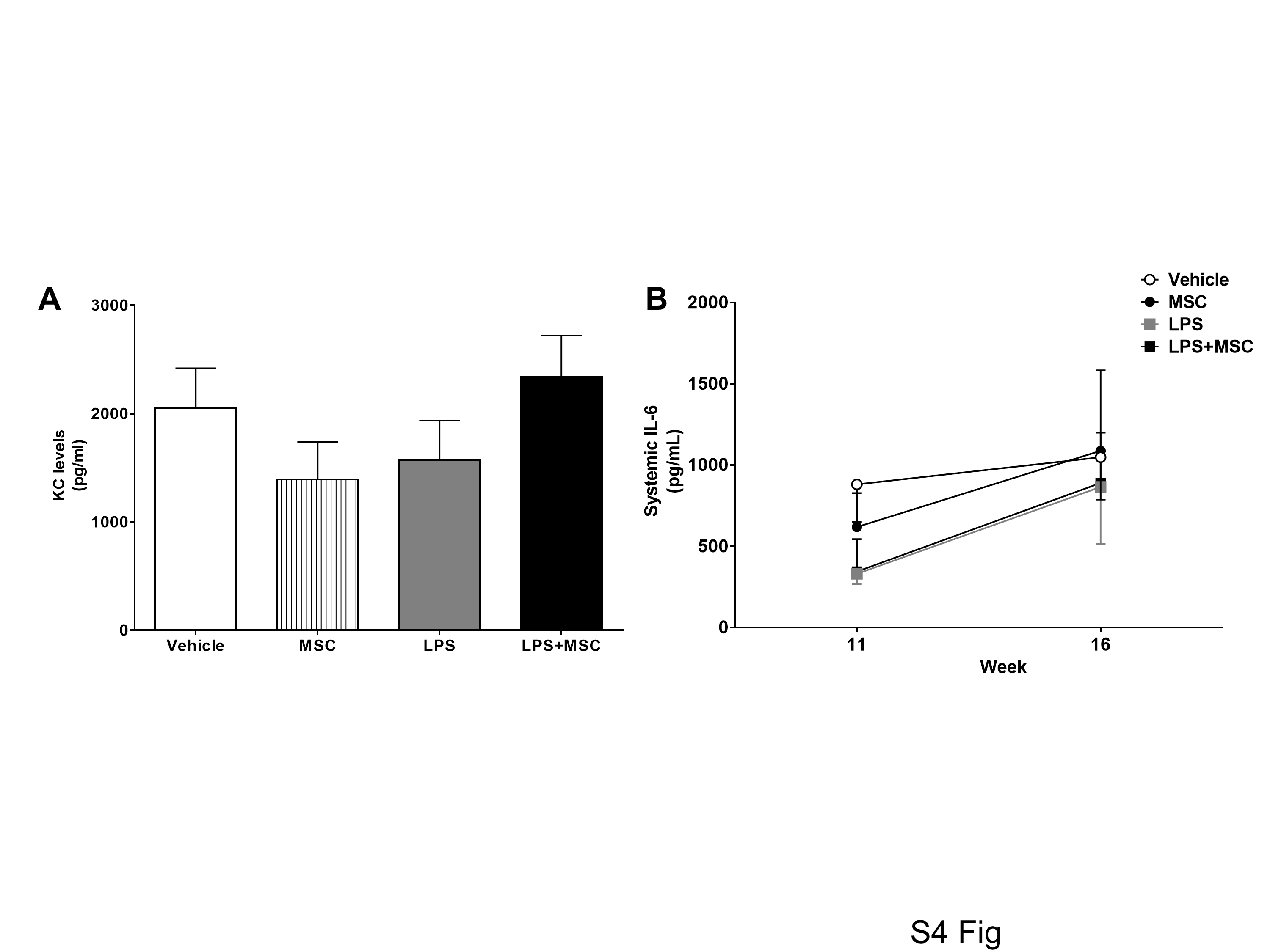

Supplement: S4 Fig — (A) Levels of KC were measured in BAL at the end of the study. (B) Levels of IL-6 were measured in plasma of mice at week 11 and week 16 (before and after MSC treatment). Data for A and B are shown as mean±SEM; n = 12–15. (TIF) [file pone.0183741.s004.tif]

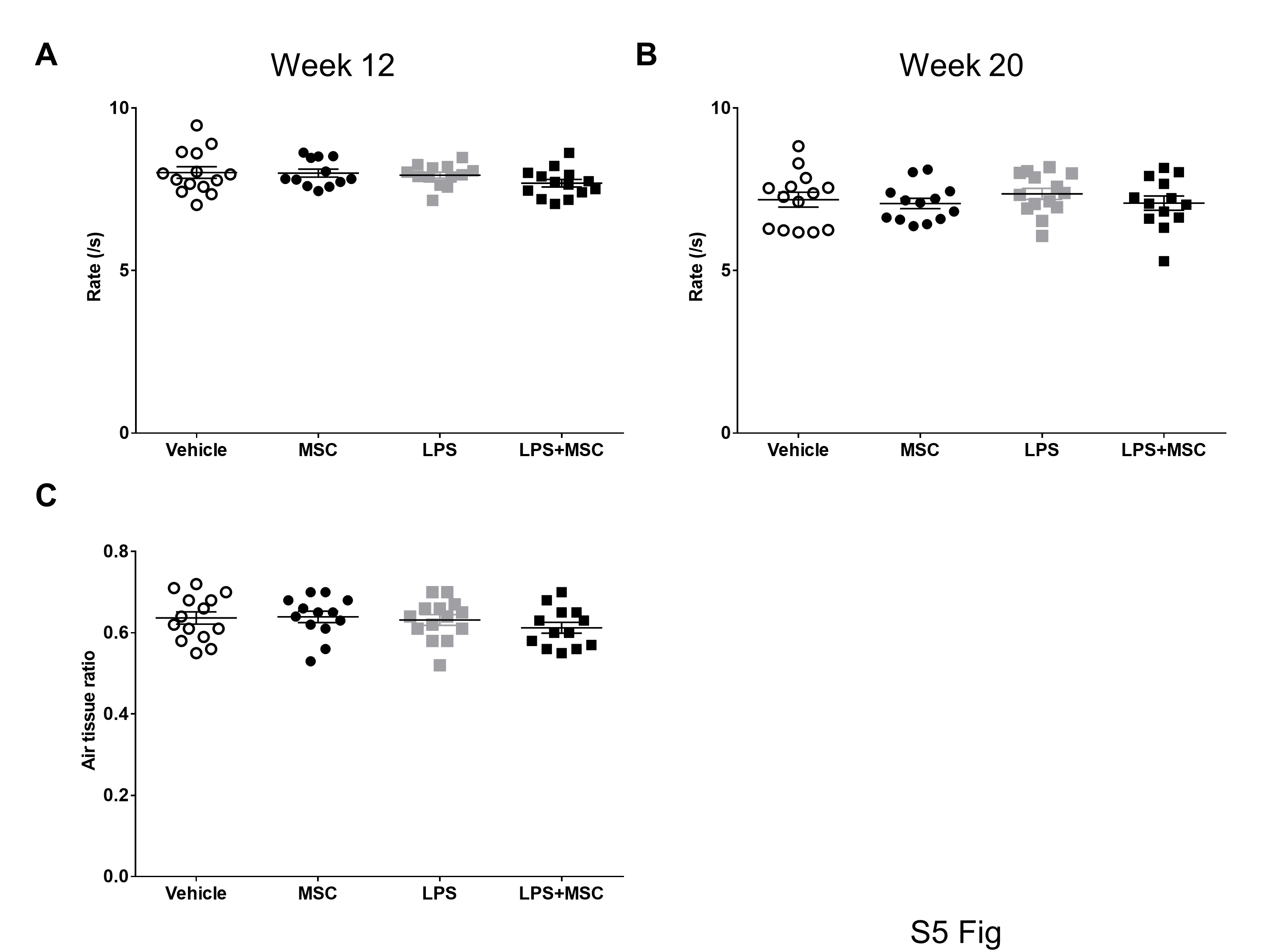

Supplement: S5 Fig — E3L mice were intranasally instilled with vehicle or 10 μg LPS twice weekly during 20 weeks WTD feeding. Mice received vehicle or 0.5x10^6^ MSC in week 14, 16, 18 and 20. Respiratory rate was measured at 12 and 20 weeks (A and B) using non-invasive whole body phlethysmography. Air-tissue ratio was assessed by morphometric assessment (C). Data are shown as mean±SEM; n = 12–15. (TIF) [file pone.0183741.s005.tif]

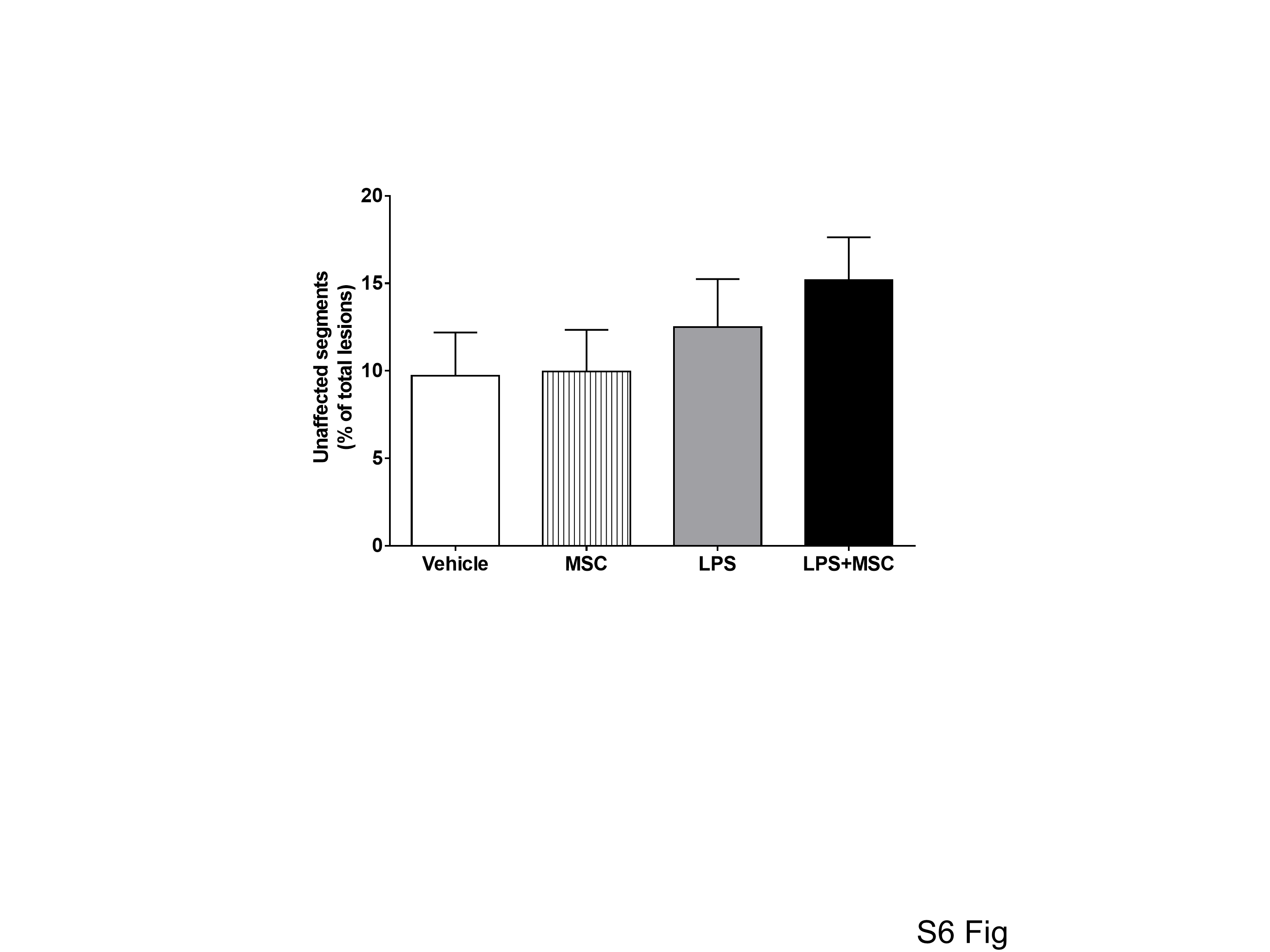

Supplement: S6 Fig — E3L mice fed a WTD were intranasally instilled with vehicle or 10 μg LPS twice weekly during 20 weeks WTD feeding. Mice received vehicle or 0.5x10^6^ MSC in week 14, 16, 18 and 20. Hearts were isolated and fixed in phosphate-buffered 4% formaldehyde and processed for paraffin embedding. Numbers of unaffected segments were determined. Data are shown as mean±SEM; n = 12–15. (TIF) [file pone.0183741.s006.tif]
